# The Impact of Iron‐Containing Particulate Matter on the Chemical Degradation of Cellulose Acetate in Collections

**DOI:** 10.1002/cplu.70265

**Published:** 2026-10-29

**Authors:** Francesca Azolini, Katherine Curran, Simoní Da Ros, Adam Gibson

**Affiliations:** ^1^ Medical Physics and Biomedical Engineering University College London London UK; ^2^ Institute for Sustainable Heritage University College London London UK; ^3^ WMG University of Warwick Coventry UK

**Keywords:** cellulose acetate, deacetylation, gas chromatography mass spectrometry, iron‐based catalysts, particulate matter

## Abstract

Particulate matter (PM) can interact with environmental factors, contributing to the chemical and physical degradation of cultural heritage materials. Although the influence of PM on polymeric materials has been widely reported—often linked to degradation in both functional and aesthetic properties—its specific impact on chemical stability remains insufficiently explored, necessitating further study to elucidate potential implications. In particular, the impact of PM on degrading cellulose acetate (CA) remains unclear, especially in storage environments employing open containment and controlled ventilation which may inadvertently promote PM accumulation. Previous studies on cellulose fibers identified a correlation between higher iron concentrations in PM and aggravated chemical degradation. Focussing on CA, this paper investigates the impact of PM on CA degradation, with particular emphasis on the role of iron in mediating chemical instability. Degradation was assessed through changes in surface appearance, degree of substitution, plasticizer content, VOC emissions, weight loss, and spectral changes in artificially aged and unaged plastic samples. Analytical techniques included scanning electron microscopy, Fourier‐transform infrared spectroscopy, thin‐film solid‐phase microextraction gas chromatography/mass spectrometry, and visible spectrophotometry. Findings indicate that iron in PM can accelerate deacetylation of CA. These results have implications for refining conservation strategies and mitigating risks associated with historical CA objects in both displayed and storage conditions.

## Graphical abstract

Artificial particulate matter with high iron content and varied iron speciation accelerates cellulose acetate degradation, increasing chemical instability during aging. These findings identify a previously underexplored deterioration pathway and demonstrate that airborne pollutant composition can critically influence the preservation of historical plastics within collections.

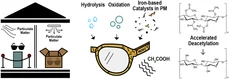

## 1 Introduction

Since the mid‐19th century, plastics have had a remarkable impact on human history. They are an integral part of our heritage and are widely found in galleries, libraries, archives, and museums (GLAM). Plastics are susceptible to degradation through a variety of processes. Environmental conditions—such as exposure to light, humidity, heat, oxygen, microbial activity, and contaminants—can initiate complex degradation pathways unique to each type of plastic. Despite extensive research aimed at understanding their characteristics, properties, degradation processes, and intervention strategies, the long‐term preservation of these materials remains a significant challenge [1, 2, 3, 4].

Generally, the impact of air pollution and the role of particulate matter (PM) on outdoor heritage objects [5, 6, 7] and the deposition/adhesion of dust particles to indoor surfaces [8, 9, 10, 11, 12] have been investigated. In the context of modern materials, some studies [3, 13, 14] have shown that dust and ingrained dirt can compromise both the aesthetic appearance and perceived significance of plastic objects in collections, particularly when compounded by ongoing degradation processes such as color change, plasticizer exudation, physical damage, and distinctive smells. On the other hand, increased chemical degradation of cellulose‐based materials has been shown to be associated with multicomponent dust [15], where aggravated effects seem to be correlated with the presence of transition metals acting as catalysts [16, 17], and the surface concentration of iron is associated with a reduction in the degree of polymerization [18].

Despite growing awareness of the detrimental effects of airborne particulate contaminants on the integrity and value of plastic artifacts in display and storage environments, the underlying chemical interaction mechanisms remain poorly understood. While preventive maintenance and cleaning protocols are commonly employed to manage PM from both indoor and outdoor sources, the extent to which PM contributes to the physical and chemical degradation of plastics, specifically cellulose acetate (CA), has not been properly examined. This study aims to investigate the role of PM in accelerating the chemical degradation of CA artifacts.

CA is one of the oldest and least stable thermoplastics found in collections, having been first prepared in 1865 [19]. The discovery of suitable plasticizers in 1927 led to the introduction of cellulose triacetate films, also known as “safety film”, widely adopted from the 1950s to 1980s as a safer alternative to cellulose nitrate (celluloid). CA was the main thermoplastic molding material until the “poly era” around 1940–1950, used in the mass production of goods such as toys [13], buttons, tool handles, camera bodies, car steering wheels, combs, etc. Even today, its applications vary from textiles [20] to plastic films and objects, such as spectacle frames and cigaret filter tows [21, 22]. As a result, CA is commonly found in GLAM collections in the form of textiles, plastic objects [23, 24], photographic, and cinematographic films.

CA is a semi‐synthetic polymer produced by the acetylation of cellulose, where hydroxyl (─OH) groups are substituted with up to three acetyl groups. The degree of substitution (DS) describes the average degree of acetylation or, more precisely, the average number of ─OH groups replaced by acetyl groups, which are then classified as diacetate (DS ≃2.2−2.7) or triacetate (DS ≃2.7−3) [25]. In addition, plasticizers are usually added to CA during processing to lower the glass‐transition temperature of the polymer and increase its flexibility [26, 27], along with stabilizers and additives to enhance appearance, durability, and performance. The heterogeneity of the composition poses a substantial obstacle for preservation efforts.

CA objects are unstable due to their inherent tendency to degrade through deacetylation. This process involves the reaction between moisture and acetyl side chain groups, leading to the production of acetic acid (AcOH) and the replacement of acetyl groups with ─OH groups, gradually converting CA back to cellulose. Acids released during this reaction further catalyze ester hydrolysis, resulting in an autocatalytic process as well as accelerating the degradation of nearby objects [2, 28]. According to recent models, the autocatalytic effect could significantly reduce the lifespan of CA objects, even under environmentally controlled conditions [29]. Plasticizer loss leading to embrittlement is another common degradation process in CA [27, 30, 31]. While the acceleration of CA degradation due to moisture and elevated temperatures has been documented [32, 33], there is currently no information on the role of PM in CA degradation pathways.

AcOH is widely reported as an indoor pollutant in heritage collections [34, 35]. Vapor‐phase concentrations of AcOH tend to increase in enclosed environments—such as storage and small‐volume display cases [36], with seasonal fluctuations influenced by temperature [37, 38]. Strategies such as lowering temperature and humidity [32, 39, 40], combined with proper ventilation [29], can mitigate such degradation effects by slowing the reactions and preventing the accumulation of AcOH. However, this strategy carries the potential risk of contamination by organic and inorganic atmospheric compounds contained in PM, whose gradual accumulation may be overlooked. Although this work does not aim to investigate AcOH as a pollutant, its capacity to exacerbate CA degradation makes it relevant to the experimental design. AcOH is introduced here as a reactive species that accelerates chemical deterioration in an enclosed environment, allowing the study to probe how PM interacts with and potentially amplifies acid‐driven degradation pathways.

PM ranges from atmospheric ultra‐fine particles, with diameters around 0.01 μm, to coarse dust, with diameters up to 100 μm [41]. PM in urban areas, including GLAM storage and collections, originates from various sources, such as traffic emissions, air conditioning systems, industrial activity, construction sites, and soiling particles [10]. More specifically, PM may include particles derived from combustion (elemental carbon with metallic traces), mineral dust, sea‐salts, organic compounds, fibers [42], and microplastics [43, 44]. Urban pollution often includes transition metals such as Fe, Cu, and Cr [45]. Furthermore, airborne PM can contain a variety of iron‐bearing compounds, including oxides, salts, metallic iron, and different types of ferrites [45], which may interact differently with CA materials undergoing degradation. Metallic iron can undergo oxidation in the presence of moisture and oxygen, particularly under elevated temperatures [45]. Certain iron salts, such as FeCl_3_, have been shown to promote deacetylation of CA [46], yielding higher conversion rates to AcOH [47, 48]. Iron storage cans are also known to have a catalytic effect on the degradation of cellulose triacetate [49]. Edge et al. [50] showed that contact with iron during thermo‐hydrolytic degradation pathways leads to a significant increase in moisture regain and a rapid decrease in viscosity retention of cellulose triacetate films. However, the impact of iron‐containing PM as a potential catalytic agent in the degradation of CA has yet to be investigated.

Other authors [17, 18, 51, 52] have previously proposed a “Fenton‐like” reaction between iron ions and hydroperoxides—which may form as byproducts of aging or exposure to light/oxygen— yielding ─OH radicals that can accelerate the oxidative degradation of CA materials, as shown below:

(1) $$Fe^{2+}+\mathrm{ROOH}\to Fe^{3+}+\cdot \text{OH}+RO^{-}$$

In addition to their role in accelerating radical‐mediated degradation pathways, ${Fe}^{2+}$ and ${Fe}^{3+}$ ions can coordinate with the carbonyl groups in CA, enhancing their susceptibility to nucleophilic attack by water molecules [53]:

(2) $$\mathrm{ROAc}\;+\;H_{2}O\;\overset{{\text{Fe}}^{2+/3+}}{\to }\;\mathrm{AcOH}\;+\;\mathrm{ROH}$$

In Equation (1), *R* represents an organic group, typically a hydrocarbon chain or ring structure, that is part of the hydroperoxide molecule. After reaction, it produces other reactive species, such as ─OH radical and alkoxy ion (•OH, ${RO}^{-}$). In Equation (2) ROAc denotes the acetylated polymer unit, AcOH is acetic acid, and ROH represents the regenerated ─OH group on the polymer backbone.

This study investigates the impact of metallic iron, iron oxide hydroxide (α‐FeOOH), and iron salt (FeCl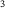) contained in variably composed PM on the deacetylation of CA. Three sources of PM soiling (one sourced from the Victoria and Albert Museum and two produced artificially—named V&A, Ar1, and Ar2, respectively) were applied to CA samples and held under accelerated aging conditions to hasten degradation (68 °C, 81% relative humidity, and in the presence and absence of additional AcOH in the air) for up to 90 days. The key distinction between Ar1 and Ar2 lies in both the concentration and the diversity of iron sources present (Table 1). PM‐free samples (referred to as Clear) were subjected to the same conditions. By measuring the degree of acetylation, off‐gassing from CA samples, plasticizer loss, mass change, and color change, we demonstrate CA chemical degradation when exposed to various PM compositions, particularly those containing iron particles. This study contributes to a further understanding of the degradation patterns of CA objects. Investigating the role of PM could reveal a previously underestimated degradation pathway in CA materials, especially in environments with poor air filtration and infrequent cleaning protocols. These findings could have significant implications for the preservation of CA materials in collections, informing targeted and context‐sensitive strategies to mitigate the interaction of PM with these artifacts.

**TABLE 1:** Relative iron contents in artificial (A) and natural (N) PM per 2 ${\mathrm{cm}}^{2}$ sample surface.

PM name (A/N)	Iron‐bearing compound	Fe, mg
V&A (N)	Unknown	Unknown
Ar1 (A)	FeOOH	0.12
Ar2 (A)	FeOOH	0.12
	FeCl 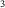	0.0184
	Metallic Fe	1.7

## 2 Results and Discussion

### 2.1 Visual Appearance

The iron‐based constituents of the PM samples employed in this study are summarized in Table 1.

Following artificial aging, samples under non‐acidic (no AcOH) conditions (Figure 1) exhibited mild warping and shrinkage, maintaining good flexibility and structural integrity. No noticeable vinegar odor was detected, suggesting that no significant deacetylation had commenced. Across all samples, discoloration is more pronounced in soiled than in Clear (PM‐free) samples, indicating a possible role of metal‐catalyzed oxidation. Specifically, a more pronounced discoloration of V&A and Ar2 samples (Figure 1b,f,j,d,h,l) and exudation, likely as a result of plasticizer migration over time, was observed. Conversely, samples subjected to artificial aging under acidic (AcOH) conditions (Figure 2) showed a loss of structural integrity, pronounced warping and shrinkage, cracking, brittleness, exudation, a pungent vinegar smell, and loss of transparency over time. These deterioration effects were especially pronounced in the Clear samples (Figure 2i) after 90 days. Curiously, when handling the samples, it was observed that those with PM coverage retained more flexibility, suggesting a potential “barrier” effect against degradation. The examination of the samples was conducted subsequent to the removal of the PM layer, which was achieved using a cloth swab.

**FIGURE 1:**
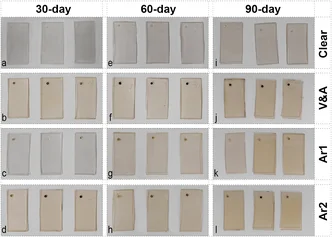
Samples after artificial aging (68 °C, 81% RH) under non‐acidic conditions. Clear (a,e,i), V&A (b,f,j), Ar1 (c,g,k), and Ar2 (d,h,l) triplicates after 30, 60, and 90 days respectively The figure depicts the condition of the samples after the removal of the PM layer.

**FIGURE 2:**
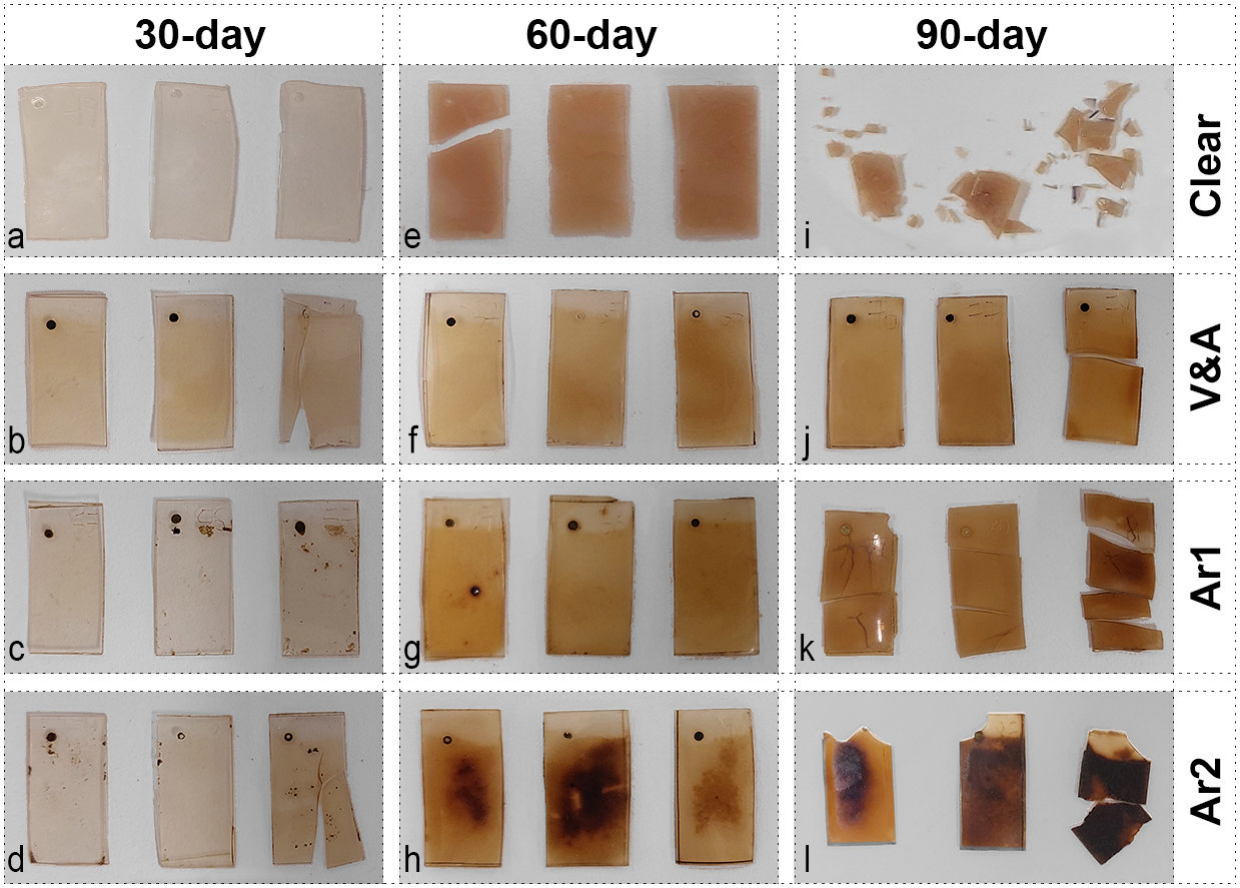
Samples after artificial aging (68 °C, 81% RH) under acidic conditions. Clear (a,e,i), V&A (b,f,j), Ar1 (c,g,k), and Ar2 (d,h,l) triplicates after 30, 60, and 90 days respectively. The figure depicts the condition of the samples after the removal of the PM layer.

Figure 3 reports SEM micrograph images that display mechanical damage and crystal aggregation occurring over time under acidic conditions. Notably, extensive crack networks (Figure 3i,j) are observed in samples with higher iron content‐PM (see Table 1) when exposed to acidic vapors. Contrary to tactile observations—which suggested improved flexibility compared to Clear samples—these samples exhibited significant mechanical damage, including crazing and delamination that were not visible to the naked eye. Interestingly, a more significant formation of crystals is observed in correspondence with mechanical damage, including breaks and cracks. These crystals manifest between CA and PM, rather than on the upper surface of PM (see Figure S1 and Supporting Information: SI.1), and are therefore mainly visible after the removal of PM. This study does not aim to characterize the crystal phases formed during aging. However, energy dispersive X‐ray spectroscopy (EDS) was performed on some detected crystals (see Figure S2 and Supporting Information: SI.1). The results do not provide definitive conclusions regarding their chemical nature, as the qualitative elemental composition identified after aging closely resembles that observed before aging despite their distinct morphology. EDS analysis suggests that the crystals formed under acidic conditions are mainly associated with calcium‐ and iron‐containing compounds. Combined with SEM observations, these results indicate the formation of new crystalline features during aging; however, the available data are insufficient to determine the specific chemical phases present. Additionally, PM residues persisted to some extent after wiping across all the samples (see Figure S3 and Supporting Information: SI.1). In general, no crystal growth was observed on V&A samples at 30 days (Figure 3a,b) nor on the Clear samples (Figure 3e,f) at 60 days. Conversely, Ar1 (Figure 3c,d) and Ar2 samples (Figure 3g,h,i,j) showed crystal formation occurring at 60 and 60–90 days, respectively.

**FIGURE 3:**
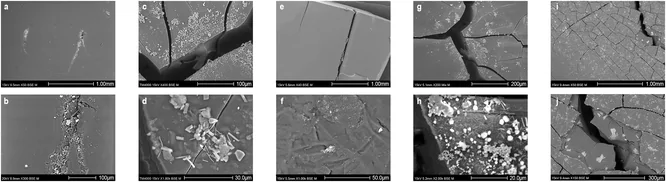
SEM micrographs depicting the condition of samples aged under acidic conditions and after the removal of the PM layer. V&A sample (a,b) (50–300X respectively) after 30 days revealing trapped solid particulate residues, indentations, but no crystals. Ar1 sample (c,d) (400–1.8kX respectively) after 60 days showing micro‐sized crystals and cracks. Clear sample (e,f) (40–1.0kX respectively) after 60 days showing no crystal formation. Ar2 sample (g,h) (200–2.0kX respectively) at 60 days revealing extensive cracks and multi‐shaped crystal formation, and at 90 days (i,j) (50–150X respectively) showing fragmented and delaminated surface appearance covered with crystals. Accelerating voltage of 15 kV and backscattered detector in medium vacuum were employed.

### 2.2 Mass Loss

Variations in the mass of CA samples were monitored to quantify material loss over time. Mass loss acts as a key indicator of material deterioration, representing the progressive breakdown of structural components. In solid CA exposed to elevated temperatures and high‐moisture conditions, this loss predominantly results from the release of water, plasticizers, and weakly bound low‐molecular‐weight volatile compounds, as well as from the cleavage of acetyl groups [27, 30, 49].

Measurements reported in Figure 4 represent the average of three measurements obtained from three independent replicates per sample. These measurements reflect the condition of the samples immediately following each aging period. Prior to weighing, all samples were stored overnight in a desiccator containing silica gel to minimize the influence of moisture regain on mass readings [54]. The samples were weighed following the removal of surface PM. Overall, as illustrated in Figure 5, there seems to be a moderately strong, statistically significant negative correlation between mass and time (*r* = −0.42, *p* ≤ 0.001).

**FIGURE 4:**
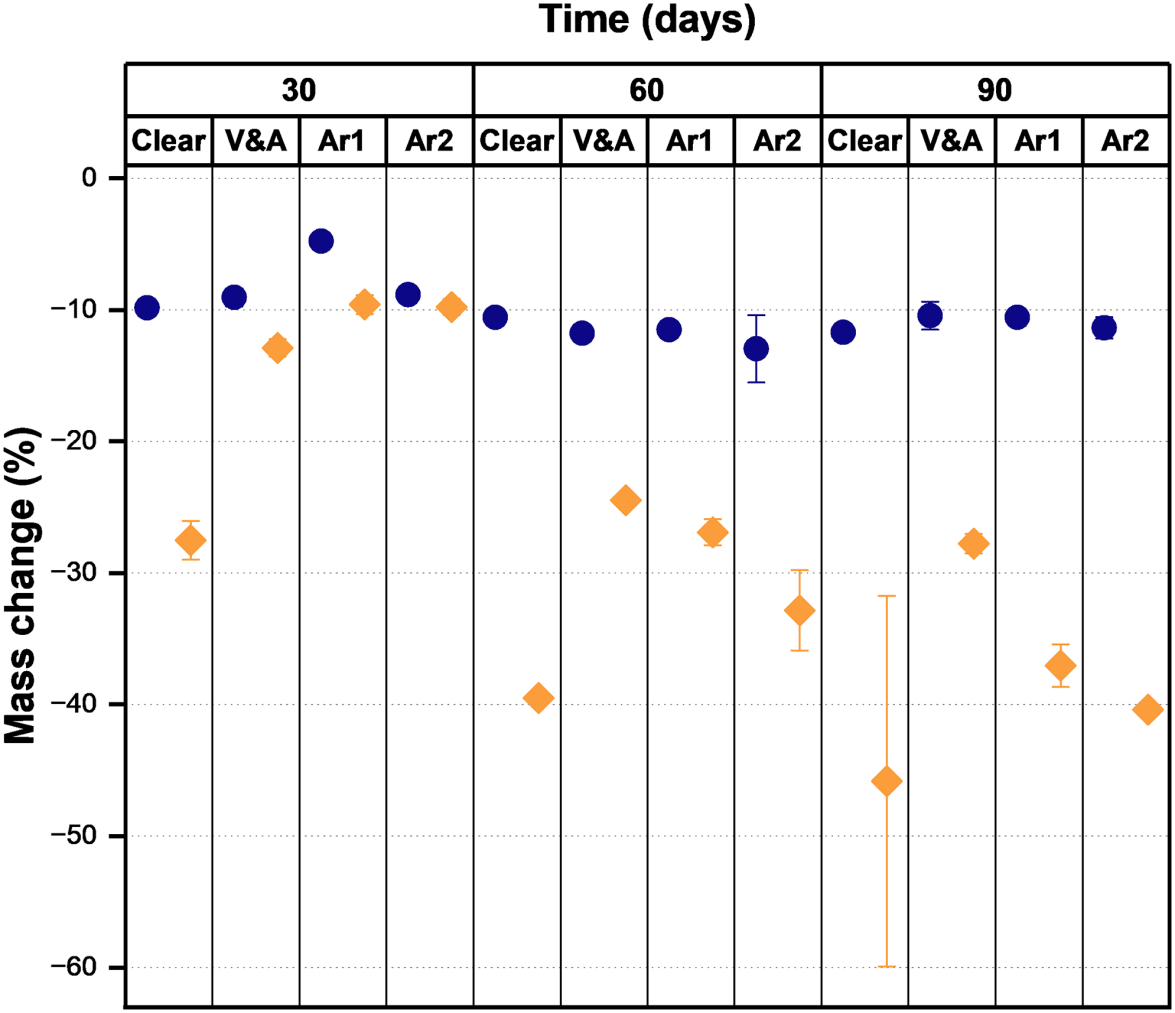
Mass change (%) of Clear, V&A, Ar1, and Ar2 samples over 90 days of aging at 68 °C and 81% RH, under non‐acidic (blue dots) and acidic conditions (orange diamonds). Data are presented as the average of three measurements performed on three sample repeats, with error bars indicating the standard deviation (*N* = 9).

**FIGURE 5:**
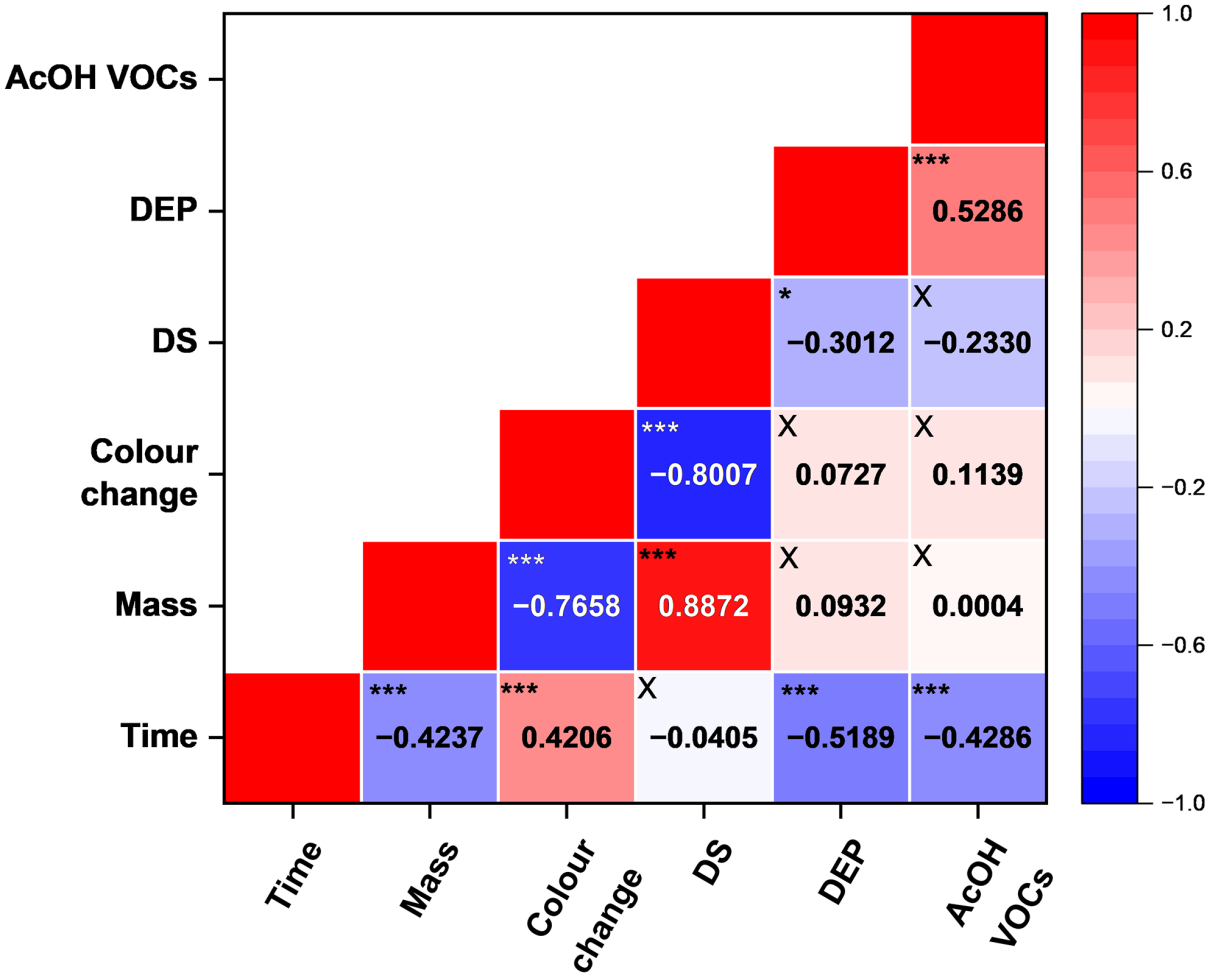
Pearson correlation plot illustrating relationships within the full sample set (*N* = 9 per sample type) between aging time, mass, color change (Δ *E* 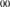), DS (1217:1030), DEP (749:601), and AcOH VOCs (peak area ratio of AcOH:MISA) following artificial aging. The color scale denotes positive (red) or negative (blue) correlations, and the intensity of the color reflects both the strength and direction of the correlation between each pair of variables (from −1 to +1). The Pearson correlation coefficient is reported together with interaction effects and their levels of statistical significance. *** = highly statistically significant effect (*p* ≤ 0.001), ** = moderately statistically significant effect (*p* ≤ 0.01), * = low statistical significance of effect (*p* ≤ 0.05), and X = no statistically significant effect (*p* ≥ 0.05).

As shown in Figure 4, in the absence of AcOH during artificial aging, all samples exhibited a comparable decrease in mass over 30–90 days. While the mass loss appears to be relatively steady across various samples over time (plateau at about −10%), an exception is offered by Ar1 at 30 days showing the lowest mass loss (−4.8 ± 0.4%), possibly due to a sample inhomogeneity. The consistency in mass loss across dirty and Clear samples over 90 days indicates that mass loss is independent of the presence or type of PM. We suspect that thermal aging in a sealed environment may have suppressed the loss of plasticizer. Some of the plasticizer that was lost could have been reabsorbed over time as the system reached equilibrium [55]. Deacetylation was not considered a contributor to mass loss here as no vinegar‐like odor was detected. This aligns with literature indicating that acetyl‐group reduction typically does not take place until after 120 days [29, 55], a result further supported by our spectroscopic data (see below).

Conversely, under induced deacetylation conditions using AcOH, all samples exhibited an incremental decrease in mass over time. Clear samples exhibited the greatest mass loss at 30 and 60 days. At 90 days, the substantial variability in mass loss (−45.8 ± 14.1%) indicates compromised measurement repeatability due to severe structural deterioration of the samples (Figure 2i). Assuming a uniform plasticizer distribution, negligible dimensional variation, and uniform PM application (≈10 mg), it can be inferred that the PM layer may act as a physical barrier, as evidenced by the severe breakdown observed in Clear samples lacking PM. This could occur by trapping acid vapors and limiting their interaction with the polymer while retaining emissions such as plasticizer, moisture, or compounds from the CA substrate. However, spectroscopic analysis suggests that the PM did not act exclusively as a passive barrier. Under acidic aging, the artificial PM formulations exhibited transient spectral changes from 30 days, including increased intensity in the O─H stretching region (3000–3500 ${\mathrm{cm}}^{-1}$), enhanced bands at approximately 3680 and 3618 ${\mathrm{cm}}^{-1}$, and the temporary appearance of bands near 1725 and 1280 ${\mathrm{cm}}^{-1}$ (see Supporting Information: SI.2) from 30 days onwards. These observations indicate interactions between AcOH and PM constituents, suggesting that both physical and chemical processes contributed to the observed degradation behavior. Its composition, however, remains critical in determining the extent of this protective effect. Indeed, higher iron concentrations appear to correlate with greater mass loss. At 60 and 90 days, among PM samples, mass loss follows the order Ar2 > Ar1 > V&A, except for V&A samples at 30 days, which appeared to be the most affected.

It seems relevant to note that significant mass loss in PM samples subjected to acidic conditions does not occur until after 60 days of the experiment. This implies that the mechanisms causing this mass loss have an induction period beginning at any point after the initial 30 days. As illustrated in Figure 4, samples exposed to AcOH exhibited substantially greater mass loss, particularly in the later stages of aging, which is likely connected to deacetylation, indicating a strong positive correlation between mass and DS (*r* = 0.88, *p* ≤ 0.001, Figure 5). For full mass change data, see Supporting Information: SI.3.

### 2.3 Color Change

In the heritage context, color change is a critical indicator not only of visual and aesthetic alteration but also of chemical change. Discoloration in plastic materials has been associated with the formation of chromophoric oxidation products [56, 57].

Figure 6 illustrates color change in samples after artificial aging, and compares it to a Δ*E* 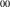 value of 1.5, interpreted as the minimum limit of variation perceptible to humans [58]. It should be noted that the PM layer was removed from the sample surface at the end of each aging period, as the interest of this study was in the physical–chemical changes of CA, without interference from PM. All samples demonstrated a consistent and progressive increase in discoloration over time (*r* = 0.42, *p* ≤ 0.001, Figure 5), ranging from subtle yellowing to extreme darkening. The amplitude of change can be related to the levels of iron (Table 1) and acid content present, with a strong negative correlation observed between DS and color change (*r* = −0.80, *p* ≤ 0.001, Figure 5). The higher the concentration and prolonged exposure to these elements, the more pronounced the discoloration becomes, as is evident when comparing discoloration between Ar1 with Ar2.

**FIGURE 6:**
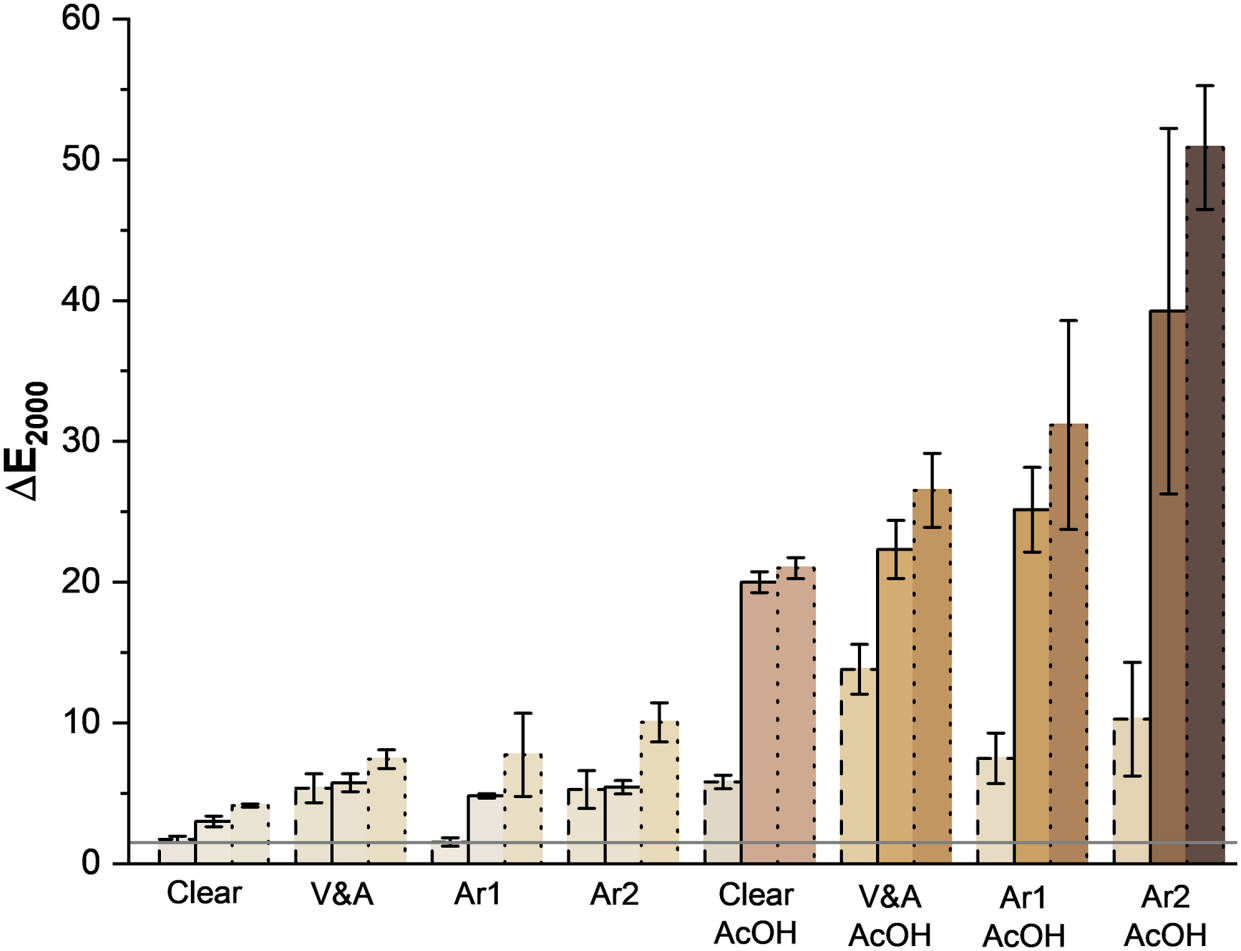
Color change for samples at 30 (dash line), 60 (solid line), and 90 (dot line) days grouped by PM type and presence or absence of acid (AcOH in this figure refers to the presence of acetic acid in the artificial aging set‐up). The gray line represents the point at which color change is perceivable to the human eye (Δ *E* 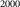 above 1.5). The colors assigned correspond to the Lab values in RGB. Data are presented as the average of three measurements performed on three samples, with error bars indicating the standard deviation (*n* = 9).

Ar2 samples at 90 days, under acidic conditions, showed the most severe color change (Δ*E* 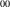 50.9 ± 4.4), which coincides with its greater loss of mass. The highest color change at 60 days is consistently attributed to Ar2 (Δ *E* 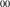 39.2 ± 12.9), where the large error may be attributed to the uneven darkening (Figure 2h), which introduces variability across the sample repeats. However, at 30 days, the most significant change among PM samples is observed in V&A (also visible in Figure 2b), which appears to support earlier findings related to its higher mass loss. Interestingly, despite being most prone to falling apart, Clear samples exhibited the least discoloration. This indicates that the mechanisms underlying discoloration and physical damage may be distinct. The presence of transition metals can catalyze oxidative reactions by increasing the production of ⋅OH radicals [59], potentially accelerating both chemical and visual deterioration. On the other hand, under nonacidic conditions, V&A exhibits the second‐highest color variation over time (also visible in Figure 1b,f,j). This suggests that unknown compositional contaminants may also significantly contribute to the discoloration process, alongside the crucial role of acid in causing discoloration.

Generally, there appears to be a moderate negative correlation between mass and color changes (*r* = −0.76, *p* ≤ 0.001, Figure 5)—samples that exhibit the most significant color change (Δ *E* 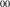 far above 1.5) also experience the greatest mass reduction. This indicates that higher iron levels, when combined with prolonged exposure to acid, are associated with more pronounced discoloration and loss of mass. As a result, the correlation implies that iron may act as a catalytic agent, accelerating oxidative and hydrolytic degradation pathways over time. Full color change data can be found in Supporting Information: SI.4.

### 2.4 Plasticizer Loss

Attenuated total reflectance Fourier‐transform infrared spectroscopy (ATR‐FTIR) has been widely used to detect the presence of plasticizers in CA objects [30, 55, 60]. ATR‐FTIR, recognized for its surface‐specific sensitivity due to its shallow penetration depth, is well‐suited for this application, as previous work [61] has shown that the loss of plasticizer diethyl phthalate (DEP) from CA primarily occurs at the surface within short exposure times.

The comparison between samples exposed to AcOH and those unexposed reveals slight variations in DEP loss. Figure 7a shows a rapid loss of plasticizer within the first 30 days across most samples, followed by a minimal change throughout the remainder of the aging period. The use of a large exposure volume—such as the desiccator container used in this study (approximately 1.9 L internal volume)—may have facilitated initial volatilization and migration of the plasticizer [60, 62]. Notably, Ar1 samples exhibited the lowest degree of plasticizer loss at the 30 days mark, consistent with corresponding mass‐loss measurements. Prolonged accelerated aging in a sealed system may have suppressed the loss of plasticizer beyond 30 days, with some content potentially reabsorbed over time as equilibrium was approached, in line with previous findings [60]. These observations also suggest that the presence of the PM layer did not significantly influence plasticizer loss.

**FIGURE 7:**
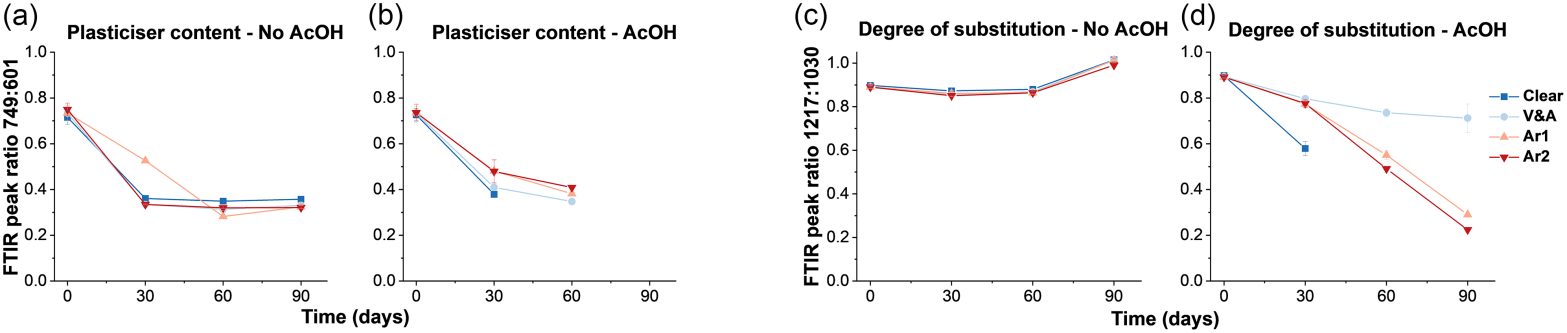
FTIR peak ratios corresponding to degree of substitution (1217:1030) and plasticizer content (749:601) for Clear, V&A, Ar1, and Ar2 samples across 90 days of aging under non‐acidic and acidic conditions. The data are presented as the average of three repeats with each sample unit measured three times (*N* = 9), with error bars representing standard deviation. Under acidic conditions, Clear (b and d) and PM (b) samples were either too fragile or lacked key spectroscopic features for analysis, resulting in the absence of 60 and 90 days calculated ratios. (a) Plasticizer content – no AcOH, (b) plasticizer content – AcOH, (c) degree of substitution – no AcOH, and (d) degree of substitution – AcOH.

Under acidic conditions (Figure 7b), Clear samples exhibited a marked reduction in DEP loss up to 30 days. Beyond this point, they were either too fragile or brittle to be analyzed, or the relevant surface‐level spectroscopic peaks had disappeared, indicating that no detectable plasticizer remained. In contrast, PM samples showed a more gradual decrease in plasticizer loss over time compared to non‐acidic conditions and exhibited a measurably reduced DEP loss compared with the Clear‐AcOH. Nevertheless, after 60 days, PM samples also lacked key spectroscopic features, precluding analysis. This suggests that the PM layer may have influenced plasticizer behavior during chemical degradation, potentially inhibiting volatilization. Similar diffusion‐barrier effects against plasticizer migration have previously been linked to emulsion layers on cellulose triacetate films [49].

Overall, PM layer may have functioned as a physical barrier, reducing DEP volatilization by limiting exposure to ambient conditions and enabling partial surface reabsorption or redeposition. Alternatively, its effects may have been negligible, with the observed trends instead governed by intrinsic material properties or environmental factors. The Pearson correlation shows a moderate negative linear relationship between DEP content and time (*r* = −0.52, *p* ≤ 0.001, Figure 5), suggesting that DEP generally decreased as aging proceeded. Further investigation into PM morphology and its interaction with plasticizer dynamics is necessary to clarify these mechanisms.

### 2.5 Deacetylation

ATR‐FTIR spectroscopy was used to monitor changes in DS and thin film solid‐phase microextraction gas chromatography mass spectrometry (TF‐SPME GC/MS) was used to detect and identify a wide range of VOCs, emitted from the samples. ATR‐FTIR is inherently limited to surface‐level measurements, and its effectiveness can be compromised under conditions of advanced degradation, where spectral resolution diminishes and overlapping bands introduce interpretive uncertainty (see Supporting Information: SI.5). In contrast, TF‐SPME GC/MS provides a complementary analytical advantage by enabling the sensitive and selective identification of both compositional elements and degradation‐related compounds. However, variability in sample homogeneity, emission rates, and matrix interactions can complicate the accurate quantification of volatiles. Overall, the combined use of TF‐SPME GC/MS and ATR‐FTIR thus provided a more comprehensive understanding of CA degradation.

#### 2.5.1 FTIR Analysis

Figure 7c shows the peak ratios corresponding to DS over 90 days of artificial aging. As expected, under non‐acidic conditions after 30 and 60 days, only a slight reduction in DS is observed. However, at the 90 days mark, an increase in DS is observed. The band at 1217 ${\mathrm{cm}}^{-1}$ became equal to or more intense than the band at 1030 ${\mathrm{cm}}^{-1}$, indicating an increase in the concentration of C─O─C ester bonds (see Figure S6 and Supporting Information: SI.6). A plausible explanation for these observations is that oxidative degradation processes may introduce aldehyde and carboxyl groups, which can react with ─OHs, forming esters [63]. An additional contributing process could be ring‐opening reactions, which would reduce the intensity of the C─O─C pyranose band at 1030 ${\mathrm{cm}}^{-1}$. Overall, these results clearly demonstrate that, in the absence of acid, chemical changes to CA occur very slowly, suggesting that the acid added to the experimental setup played a crucial role in accelerating chemical changes within a shorter time frame.

In contrast, over the 90 days aging period under acidic conditions (Figure 7d), all samples exhibited a gradual reduction in DS, with the most pronounced decrease in the Clear samples, followed by Ar2. Notably, for the Clear samples, it was not possible to calculate the DS peak ratio beyond 30 days due to the severity of degradation under AcOH conditions, which resulted in the loss of the distinctive spectral peaks (as visible in Figure S5 and Supporting Information: SI.6). However, the deacetylation trend could also be inferred by a reduction in intensity of the acetyl carbonyl band at 1730 ${\mathrm{cm}}^{-1}$, accompanied by an increase in the ─OH band around 3480–3300 ${\mathrm{cm}}^{-1}$. In the context of ATR‐FTIR analysis, these spectral regions were excluded from calculated intensity ratios because multiple degradation pathways may coexist (see Supporting Information: SI.5), especially around 1730 ${\mathrm{cm}}^{-1}$ where DEP peak may overlap (see Figure S5 and Supporting Information: SI.6). Nonetheless, an examination of the peak intensities associated with the ─OH group (Figure 8) suggests a discernible trend over time. Given that the samples were dried at each aging interval, the ─OH peaks were interpreted as indicators of degradation rather than moisture. It can be seen that the rate of deacetylation follows the order Clear > Ar2 > Ar1 > V&A, which is confirmed by the calculated ratios (Figure 7d).

**FIGURE 8:**
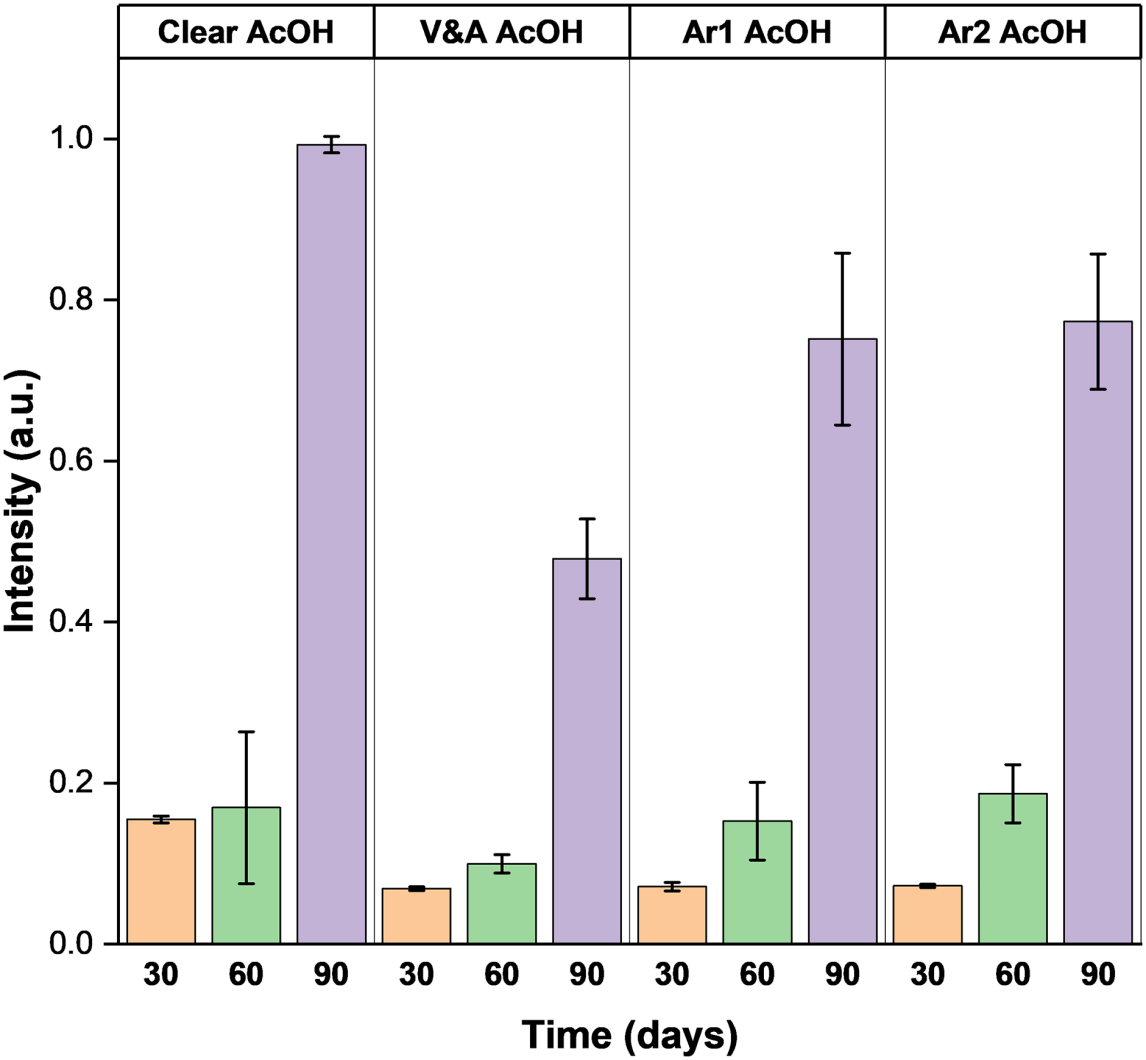
Normalized FTIR peak intensities of hydroxyl groups (band range 3480–3300 ${\mathrm{cm}}^{-1}$) for Clear, V&A, Ar1, and Ar2 samples over 90 days period under acidic conditions. Data are presented as the average of three measurements performed on three sample repeats, with error bars indicating the standard deviation (*N* = 9). These results highlight a marked increase in the ─OH group over time, with Clear and Ar2 showing the highest intensities.

Figures 7d and 8 demonstrate that the presence of PM may inhibit deacetylation, particularly in its initial phases, while also indicating that the composition of PM induces a distinct response in the surface‐level chemical changes. Table 1 displays the relative iron contents of both artificial PMs. The findings suggest that a higher iron content does promote deacetylation. However, the difference between deacetylation in Ar2 and Ar1 samples is not that significant.

#### 2.5.2 VOCs Emission

In this study, AcOH served as a marker of degradation, signifying both the breakdown of the polymer and, when the samples were aged under acidic conditions, the acid absorbed by the plastic during the experiment. Vapor‐phase concentrations of AcOH within the headspace of the desiccator were not measured directly. Instead, the primary objective was to quantify the off‐gassing phenomena occurring from the CA substrates after exposure to aging conditions.

All samples’ extractions were collected within a single month, regardless of when they were removed from the aging setup, to reduce potential variability arising from TF‐SPME membrane degradation, column sorption changes, or contamination of the ion source—factors known to affect long‐term GC/MS performance. Despite efforts to minimize the time between sample preparation and analysis, some loss of volatile compounds during handling and inherent variations in sample preparation must be considered.

The repeatability of VOC emission measurements was generally moderate, as reflected by the error bars (Figure 9); however, significant differences between samples remained discernible. The relative standard deviation of VOC emissions from CA samples was notably high compared to the respective inter‐day variation of internal standard peaks (see Supporting Information: SI.7), suggesting poor reproducibility. Significant variability aligns with previous observations of VOC analysis from solid plastic samples [64], which may be attributed to differences in surface area in particularly damaged samples, in addition to the sampling process and instrumental factors.

**FIGURE 9:**
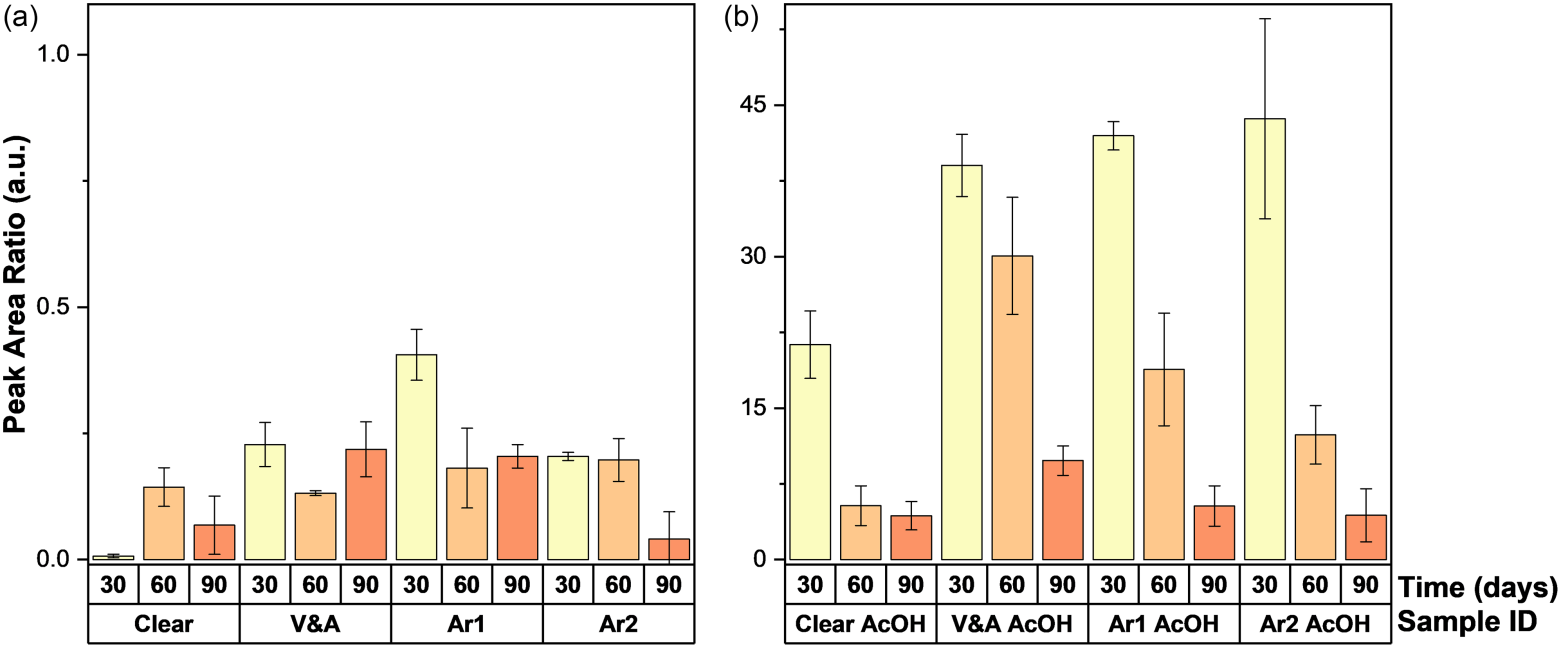
Peak area ratio (peak area of AcOH:peak area of MISA, see Experimental section for details) of acetic acid emissions over 90 days artificial aging period for different sample types, under non‐acidic (a) and acidic (b) conditions. The data are presented as an average of three samples repeats (*N* = 3), with error bars representing standard deviations.

Under non‐acidic conditions (Figure 9a), no distinct trend of AcOH emission was observed over the 90 days period, despite the average emissions across all PM samples being higher than those from Clear ones. However, no correlation was found between the amount of acid emitted and the iron content.

Overall, under acidic conditions (Figure 9b), Clear samples emitted the least, whereas PM samples appeared to retain more acid. Moreover, AcOH emission levels were observed to decrease over time, which contrasts with findings reported in previous studies [28, 65, 66]. Notably, during the first 30 days of artificial aging, samples exposed to PM exhibited a comparable increase in acid emission. However, from 60 to 90 days, V&A samples retained and emitted a greater amount of AcOH. This indicates that, under deacetylation conditions, more extensively deacetylated samples (Figure 7d) generally retain less acid. These observations support a negative correlation between DS and AcOH emissions (*r* = −0.23, Figure 5) under the tested conditions. This suggests that most acetyl groups in the Clear samples have already been cleaved, leaving less acid in the sample. In contrast, in the Ar1 and Ar2 samples, AcOH was likely absorbed by the soiling and transformed into crystalline phases (Figure 3d,h,j), which subsequently decrease its volatility [67]. Therefore, establishing a direct correlation between higher iron content and acidic emissions remains complex due to multiple interacting processes.

## 3 Conclusion

This study examines the impact of PM, with a specific focus on the contribution of iron to the chemical degradation of CA. Considering the extensive presence of this material in heritage collections and the significant role of dust and dirt as forms of damage to plastics, it is important to elucidate these degradation dynamics to inform effective preservation strategies applicable to both exhibition and storage contexts. The proposed method investigates CA plasticized with DEP, covered with three distinctive PM formulations (V&A, Ar1, and Ar2), and subjected to artificial aging (68 °C, 81% RH, under acidic and non‐acidic conditions) for up to 90 days. The following conclusions can be drawn:

• Under non‐acidic conditions, no evidence of deacetylation was observed over 90 days; mass and DEP loss remained stable and were apparently unaffected by PM. However, color change progressed steadily and correlated with PM type, likely driven by iron‐catalyzed oxidative processes.
• Under acidic conditions, differences in chemical degradation between samples with different soilings only became apparent after 30 days of artificial aging. Clear samples consistently exhibited poorer performance overall, indicating that direct exposure to the AcOH‐rich environment was the dominant driver of deterioration.
• A generic PM layer may act as a physical barrier, slowing or altering chemical reactions by modifying exposure to moisture and AcOH. However, the PM composition influenced the degradation behavior. While samples exposed to V&A PM consistently exhibited the least severe changes across all measured parameters, samples exposed to iron‐containing artificial PM (Ar2) consistently showed the second highest degree of degradation after the Clear samples. Iron‐bearing PM was also associated with distinct surface alterations and crystalline features, indicating that reactive particulate components can contribute to local deterioration processes.
• Under severe degradation, AcOH emissions and DS exhibited opposite trends; reduced emissions (Ar2) may indicate crystal formation. Future work should refine TF‐SPME, test ground samples for improved sensitivity, and explore applications to historical materials.
• The integration of surface spectroscopy, headspace GC, and SEM‐EDS provided a deeper understanding than mass and color change measurements alone, highlighting that physical changes may not reliably reflect complex chemical degradation processes [20].
• PMs accelerate discoloration but may inhibit deacetylation—which leads to mechanical damage—likely because they retain the acids that drive the reaction.

The present study provides new insights into the degradation processes of CA in the presence of PM. The exceptionally high AcOH concentrations employed for accelerated aging likely influenced the degradation behavior observed and may have contributed to the apparent barrier effect of PM; consequently, these results should not be considered directly representative of museum environments. Under the conditions investigated, it remains difficult to determine whether the apparent barrier effect of PM or the reactivity of iron‐containing particles was the dominant factor governing degradation. Nevertheless, the study demonstrates that PM composition can influence the degradation of CA, with Ar2 associated with more pronounced deterioration than the V&A PM investigated here. While PM was not identified as the primary driver of degradation, the contrasting behavior observed between PM types highlights the importance of considering not only the quantity but also the composition of deposited PM.

These findings are particularly relevant in heritage collections, where CA objects may already be at different and often unknown stages of degradation and where the composition and reactivity of deposited PM are rarely characterized. Consequently, minimizing PM accumulation through effective air filtration and regular cleaning remains an important preventive conservation measure.

Future research should expand to cellulose triacetate and other CA variants with different thicknesses and plasticizers, while also examining the effects of fluctuating temperature and humidity. Such work will deepen our understanding of environmental interactions and support the development of more robust preservation protocols for CA‐based heritage materials in collections and storage.

## 4 Experimental Section

### 4.1 Sample Preparation

#### 4.1.1 Plasticized CA

CA with an average DS of 2.45 ± 0.05 and DEP (99.5%) were supplied by Sigma–Aldrich (Merck, Germany) and used as received. Plasticized CA samples were synthesized via solvent casting with 20 wt% DEP, as described elsewhere [26, 55]. The resultant mixture was kept under reflux for 4.5 h at 62–64 °C with continuous stirring, allowed to cool for 1 h with gentle stirring, and finally poured into a flat glass tray. Slow evaporation was allowed under a fume hood by keeping a glass lid over the sample tray at room temperature for about four days. Finally, drying was performed in a vacuum oven (150 mbar) for 120 h between 20° and 23 °C. The newly formed CA sheet was cut into regular samples (approximately 1 cm × 2 cm × 0.7 mm for an average of 136 mg) with a scalpel. The samples were stored in individual polyethylene bags at 4 °C when not in use. Three repeats of each sample were made, and three controls of each sample were always kept in the fridge for comparison and representation of an untreated condition. A total of 84 samples were prepared and analyzed.

#### 4.1.2 PM

Three types of soiling were used to model indoor PM. One naturally‐sourced (named V&A) was collected from Level 0 ‐ room 50a (Cromwell road entrance) of the Victoria and Albert Museum in London and used for the purpose of this experiment. The material exhibited a dry, powdery appearance with a dark gray coloration. It was prepared by adding 0.5 mL olive oil (Filippo Berio, USA), 1 mL mineral oil (Merck, Germany), and 50 mL petroleum ether (VWR Chemicals, UK) to 5 g of the V&A solid mixture crushed with a grinder. The agglomerated part, consisting mainly of fibers, was filtered and discarded. Considering the heterogeneity of this compound (combusted particles, mineral aggregates, fibers, potential microplastics, and others), it is difficult to determine the exact amount and composition of solid components, including Fe‐particles, intended for each sample. Nevertheless, it is believed that the use of this product is highly illustrative of a real case.

The second investigated soiling (named Ar1) was artificially prepared following a published recipe [68]. It contained 1 mL olive oil, 2 mL mineral oil, 100 mL petroleum ether, and a particulate mixture of 0.2 g carbon black (Kremer, Germany), 0.05 g α‐FeOOH (Kremer, Germany), 0.175 g silica (Sigma–Aldrich, Germany), 2 g kaolin (SLS, UK), 1 g gelatin powder from porcine skin (Sigma–Aldrich, Germany), 1 g soluble starch from wheat (Sigma–Aldrich, Germany), and 1.75 g Portland cement (Lafarge, France). Ormsby et al. (2010) represents one of the most commonly used artificial compounds to mimic atmospheric soiling in the heritage sector.

The final one (designated Ar2) is derived from Ar1, with the addition of supplementary iron sources, specifically 0.0138 g FeCl‍‍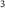 (98%) (Thermo Fisher Scientific, UK) and 0.43 g metallic iron (<10 μm) (Merck Millipore, Germany). The quantity of FeCl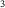 was determined based on literature [18], with the intention of significantly exceeding the threshold of 23 μg/cm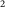 of Fe—previously shown to trigger depolymerization in cellulose‐based materials. Ultimately, the metallic iron amount was informed by authors’ previous studies. For complete description of Fe content and types of PM used, refer to Table 1.

V&A and Ar1 mixtures were stirred on a magnetic plate (VMS C7, VWR) and applied in a quantity of 400 μL per sample (two coats of 200 μL), with a Socorex Acura 825 micropipette (100–1000 μL). The same applies to Ar2, which was, however, manually mixed, as metallic iron would adhere to the magnet. The application method guaranteed a fixed and constant amount of PM on each sample. The estimated quantity of Fe in Ar1 samples is 0.12 and 1.84 mg/cm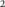 in Ar2. Artificially elevated concentrations of iron were employed to observe the degradation pathways of CA under thermo‐hydrolytic and thermo‐oxidative conditions. All samples were left to dry undisturbed and sheltered for three weeks at lab conditions (19 °C, 45% RH) before starting artificial aging. Once the experiment concluded, the samples were cleaned of the PM layer using a cotton swab to obtain data representative solely of the CA.

### 4.2 Artificial Aging

Artificial aging conditions were used to accelerate decay mechanisms, specifically deacetylation [54, 69, 70, 71]. Samples were aged in an oven (VENTI‐Line Prime, Avantor, UK) at 68 °C and 81% RH for three months, placed on sieve porcelain plates within airtight 1.9 L desiccators (Jencons Scientific Apparatus Makers, Hemel Hempstead, Hertfordshire, England), with 50 mL saturated salt solution of potassium chloride (KCl) underneath [72]. Some samples (named as “Sample‐typeAcOH”) were exposed to an aqueous solution of 13.54 wt% AcOH (glacial 100%) (Merck Millipore, Germany), prepared by adding 7 mL AcOH to the saturated KCl solution. The resulting solution corresponded to an AcOH mole fraction of approximately 0.04 in the liquid phase. Calculation of the AcOH concentration in the vapor‐phase is nontrivial due to the nonideal behavior of AcOH and its tendency to dimerize in the vapor‐phase [73]. Therefore, we relied on studies that consider AcOH activity and fugacity coefficients [74, 75, 76]. The AcOH concentration above the solution at 68 °C was calculated to be approximately 11.3 × 10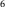 μg $m^{-3}$ (approximately $5\times 10^{6}$ ppb [37, 77]). AcOH was used at an extremely high concentration to promote detectable changes over the duration of the experiment. The concentration was selected based on previous studies within our group, in which a reduction in DS was observed within 30 days. This accelerated aging condition was designed to increase the rate of degradation and was not intended to simulate real environmental conditions, where AcOH concentrations are generally reported in the range of 40–280 ppb [37]. The desiccators were kept closed for the entire duration of each aging period to saturate the volume of air and reach equilibrium. Triplicate samples were withdrawn for degradation characterization at 30, 60, and 90 days intervals and allowed to stabilize at room temperature for about an hour, then placed in a dried desiccator with silica gel overnight before analysis. Humidity and temperature in the desiccators were monitored using TinyTag Ultra2 sensor (±0.02 °C).

### 4.3 Scanning Electron Microscopy‐EDS

Scanning Electron Microscopy (SEM) was performed in a field emission Hitachi Tabletop microscope TM4000 with a backscatter detector in medium‐vacuum mode to accommodate both conductive and nonconductive specimens, such as CA samples and PM residues. An accelerating voltage of 15 kV was mostly used, with adjustments made on the specific requirements. Images were captured at various magnifications, ranging from 40× to 2000×. Acquired images were processed with TM4000 software (version 1.6.2.0). Elemental mapping was performed with a QUANTAX XFlash energy dispersive X‐ray spectrophotometer system (Bruker) with silicon drift detector and a scanning control unit. Data analysis was processed with ESPRIT compact software.

### 4.4 Spectrophotometer

Color measurements were taken after each time intervals of artificial aging using a visible spectrophotometer (Ci62x, X‐rite, US) with illuminant D65, standard observer angles 2°>, specular component included. Three repeated measurements were taken and averaged. Color space was measured using the CIELab [78], where perceived colors are described by L* referring to lightness (black to white), a* for green to red, and b* for blue to yellow. Differences in color before and after each aging period were calculated in accordance with CIEDE 2000 (Δ *E* 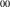) [79, 80].

### 4.5 ATR‐FTIR

Samples were kept at room temperature in a desiccator with silica gel (Fisher Chemical, USA) overnight before performing the analysis [54] to avoid moisture accumulation, which could compromise the interpretation of the ─OH band. Infrared spectra were collected from samples before and after artificial aging, using a ATR‐FTIR (Alpha‐P, Bruker) equipped with diamond cell, over a wavenumber range of 4000–400 ${\mathrm{cm}}^{-1}$. Thirty‐two scans were collected and averaged using a spectral resolution of 4 ${\mathrm{cm}}^{-1}$. The spectra were baseline corrected by fixing the average absorbance between 2200 and 2000 ${\mathrm{cm}}^{-1}$ at zero before the extraction of individual band intensities. Spectra were averaged, baseline corrected and normalized using MATLAB2023b. Changes in DEP concentrations were identified using the ratio between the maximum absorbance intensity around 749 ${\mathrm{cm}}^{-1}$, corresponding to the CH benzene band (phthalate), and 601 ${\mathrm{cm}}^{-1}$, representing the C─C─C backbone of the cellulose ring (CA) [55]. The 601 ${\mathrm{cm}}^{-1}$ peak was used as an internal standard, as it appears to remain unaffected by the concentration of plasticizer and the hydrolysis process [81]. The calculation of DS change was based on the ratio between the maximum absorbance intensity of the C─O ester vibration, observed around 1217 ${\mathrm{cm}}^{-1}$, and the C─O─C vibration from the CA backbone around 1030 ${\mathrm{cm}}^{-1}$, assumed to remain unaffected during this experiment. Peak intensities of ─OH (3480–3300 ${\mathrm{cm}}^{-1}$) bands were also studied. Peak ratios were computed exclusively when the intensities of the peaks of interest were distinctly discernible, thereby minimizing interpretation errors. Three measurements at random locations were taken on each replicate, resulting in an average of nine measurements per sample type. Data visualization was conducted in Origin Pro 2025 (version 64‐bit 10.2.0.188).

### 4.6 TF‐SPME GC/MS

A Perkin Elmer Clarus 590 gas chromatograph equipped with a Perkin Elmer Clarus SQ8S mass spectrometer was coupled to a Markes Centri 360 system for sample thermal desorption. Data collection was controlled via the Perkin Elmer TurboMass (version 6.1.2.2048) software and the CENTRI via the Markes Instrument Control (version 2.1) software. The VOCs identification was performed using the NIST Mass Spectra Library V2.3, and data analysis (peak area integration and data visualization) with OriginPro 2025 (version 10.2.0.188).

A VOCOL column (Supelco, fused silica capillary column, 24 154) was used (60 m in length and 0.25 mm × 1.5 μm in diameter) to separate the VOCs using the GC oven program as follows: initial temperature of 40 °C (held for 5 min), ramp rate of 5 °C/min to 235 °C (held for 17 min), giving a total run of 61 min. The system was configured with a splitter port (5 mL/min He‐flow supplied) inside the GC, with two transfer lines to an odor port and the MS. However, olfactory detection was not utilized in this study. The carrier gas (He) was maintained at a head pressure of 27 psi. During the GC run, the flow rate was approximately at 1.5 mL/min at the midpoint. Mass spectra were collected under electron ionization mode at 70 eV and recorded from *m*/*z* 45–300 with a scan time of 0.5 s, inter‐scan time of 0.04, and a solvent delay of 1 min.

Desorption of the TF‐SPME membranes was operated with a desorption time of 10 min at 230 °C and a trap flow of 50 mL/min. The trap purge was maintained for 1 min with a 50 mL/min purge flow. The VOCs were trapped and desorbed onto a focusing cold trap for material emission (Markes International, UNITY‐xr TD‐100, U‐T12ME‐2S) with a maximum heating rate up to 300 °C. The TF‐SPME membranes were reconditioned at 260 °C for 20 min with a 50 mL/min split flow before each sample run. Furthermore, blanks of the TF‐SPME were performed using the desorption method described above to confirm the absence of contaminants and thus assess the effectiveness of the reconditioning process.

The sampling extraction was performed in headspace mode [82, 83], which is based on the absorption of the analytes on a fiber coating of a thin film placed in the sample’s headspace volume. The extraction of VOCs was done on TF‐SPME with a polydimethylsiloxane/hydrophilic lipophilic balanced (PDMS‐HLB) coating membrane (C‐TF‐PHLB‐5, Markes International), which makes a suitable affinity for polar and nonpolar compounds. The TF‐SPME membranes were exposed to CA samples (1 cm × 2 cm × 0.7 cm), with an approximate mass of 107 mg per degraded sample, for 1 h at 60 °C [64] in 20 mL, 18 threaded headspace sample vial (Perkin Elmer, N9306242) with a headspace cap (18 mm metal screw cap blue sil/white PTFE, Perkin Elmer, N6356475). The TF‐SPME was suspended in the vials using a stainless‐steel split pin; thus, there was no direct contact between the sample and the thin film (see Figure S7 and Supporting Information: SI.7). Immediately after extraction, TF‐SPME fibers with analytes were retracted from the sample vial and transferred to desorption tubes containing Tenax TA sorbent (C3‐CAXX‐5304, Markes) for analysis, as per above.

To account for instrumental variability and ensure consistent quantification of volatile compounds of interest over the period of study, peak areas of target analytes were normalized to a reference compound and their retention times calculated (see Supporting Information S7). A MISA Group 17 Non‐Halogen Organic Mix was purchased from Sigma–Aldrich (48133, Merck) and used as an external standard [64]. It contains 2000 μg/mL each of benzene, ethylbenzene, styrene, toluene, and *ortho*‐, *meta*‐ and *para*‐xylene in methanol. 1 mL of this was diluted up to 50 mL in methanol. On each day of analysis, one TF‐SPME membrane was exposed to 1 mL of this solution for 1 min before being analyzed in the GC/MS. A ramped temperature program was followed where the initial temperature of 50 °C was held for 5 min, ramp rate of 10°/min to 100°, then 5°/min to 200°, and then 2°/min to 220° held for 20 min for a total time 31.5 min. Mass spectra were collected under electron ionization mode at 70 eV, with mass recorded from 45 to 300 *m*/*z*, a scan time of 0.4 s (inter‐scan delay of 0.05), and a solvent delay of 2 min. This provided daily data on instrumental and column drift, along with the performance of the TF‐SPME membranes. AcOH, a notable volatile compound examined in this study, was identified by its characteristic peak at *m*/*z *= 60. The changes in two peak areas corresponding to benzene (16.1 min) and toluene (20.4 min) were monitored in intra‐day and inter‐day measurements to account for variability and repeatability (Supporting Information S8).

The standard protocol included the analysis of the MISA standard together with the analysis of 9 samples, each alternating with a GC blank to avoid cross‐contamination, the reconditioning of 10 TF‐SPME membranes, and then the analysis of 10 blanks (empty tubes containing only membranes). The peak areas of the analytes were normalized relative to MISA using Origin Pro 2025.

### 4.7 Statistical Methods

Pearson correlation analysis was conducted to examine linear relationships between key quantitative variables measured throughout the experiment. The analysis aimed to determine the strength and direction of associations between changes in physical and chemical material properties—namely mass change (%), color change (Δ *E* 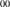), DS reduction, plasticizer loss, and AcOH VOC emissions—and the experimental conditions of interest (time, PM type, and presence/absence of acid during artificial aging). Pearson’s correlation coefficient (r) expresses the strength and direction of a linear relationship between two continuous variables, with values ranging from −1 (perfect negative linear association) to +1 (perfect positive linear association), while *r* = 0 indicates no linear relationship. It is acknowledged that combining data from acidic and non‐acidic conditions may produce more generalized correlations; however, this approach was considered necessary because the addition of AcOH was essential for inducing measurable deacetylation within the experimental time‐frame.

To establish the statistical significance of the results, we employed a combination of paired *t*‐tests associated with Pearson’s r. Statistical significance was set at 0.05, and exact *p*‐values were reported. Correlations with *p* < 0.05 were interpreted as statistically significant, indicating evidence of a non‐zero linear association between the variables. This combined approach allowed us to distinguish meaningful trends from those that may have arisen due to limited data.

## Funding

This work was supported by the Engineering and Physical Sciences Research Council (EP/R513143/1 and EP/T517793/1).

## Conflicts of Interest

The authors declare no conflicts of interest.

## Supporting information

Supplementary Material

## Data Availability

The data that support the findings of this study are available in the Supporting Information of this article.
