# Supplementary material for: The Impact of Iron‐Containing Particulate Matter on the Chemical Degradation of Cellulose Acetate in Collections
Source: Chempluschem. 2026 Oct 29;91(11):e70265. doi: 10.1002/cplu.70265 (PMC13719889; doi:10.1002/cplu.70265)
Supplement: Supplementary file 1 [file CPLU-91-e70265-s001.pdf]

SUPPORTING INFORMATION

Francesca Azolini, Katherine Curran, Simoní Da Ros, Adam Gibson

October 26, 2026

1 SI.1: SEM-EDS images

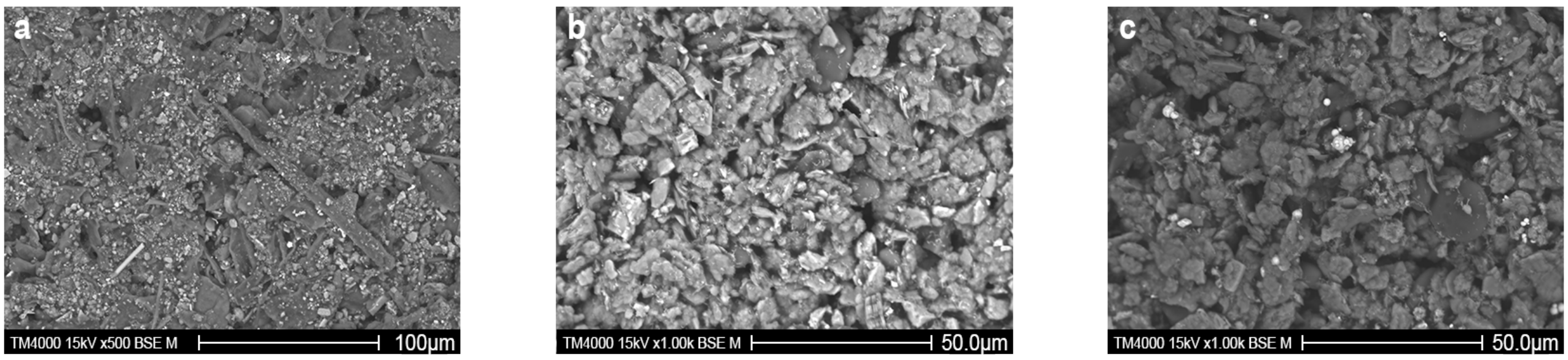

Figure S1: SEM micrographs showing absence of surface crystals on three PM types–V&A **(a)** (500X), Ar1 **(b)** (1.0kX), and Ar2 **(c)** (1.0kX)– prior to wiping and following 90 days of ageing under acidic conditions.

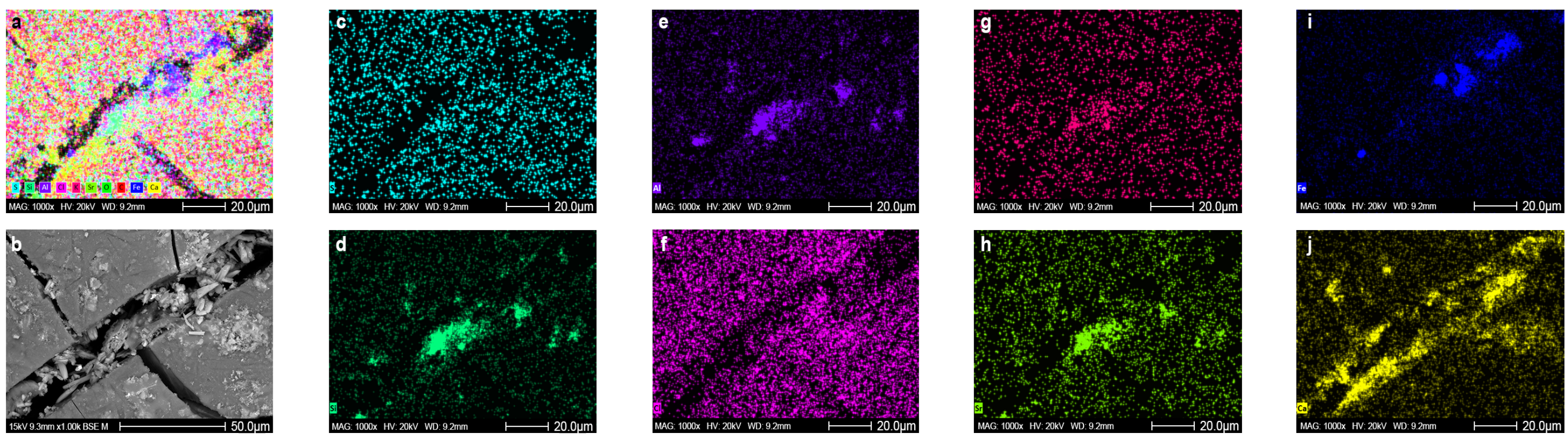

Figure S2: SEM-EDS micrographs **(a-b)** (1.0kX) of Ar2 sample after 90 days of exposure to acidic conditions and PM removal. Crystal identification was attempted via EDS, revealing the presence of S **(c)**, Si **(d)**, Al **(e)**, Cl **(f)**, K **(g)**, O, C, Sr **(h)**, Fe **(i)**, and Ca **(j)**. Oxygen and carbon are excluded from the coloured EDS maps due to probable interference from the plastic substrate. SEM-EDS analysis indicates the presence of calcium and iron compounds in crystalline form after prolonged ageing under acetic acid conditions.

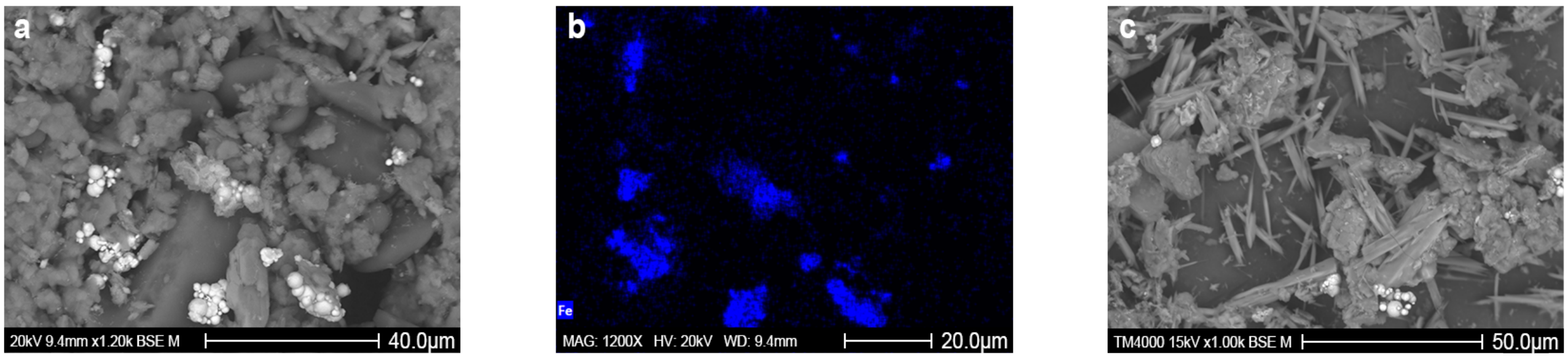

Figure S3: SEM micrographs of Ar2 sample before ageing treatment **(a)** (1.2kX) reveal the presence of spherical particles identified as metallic iron **(b)** (1.2kX). After 90 days of exposure to acidic conditions and wiping, the same sample still exhibits some residues of metallic iron particles **(c)** (1.0kX), now accompanied by newly formed crystalline structures that were absent prior to ageing.

2 SI.2: FTIR of PMs

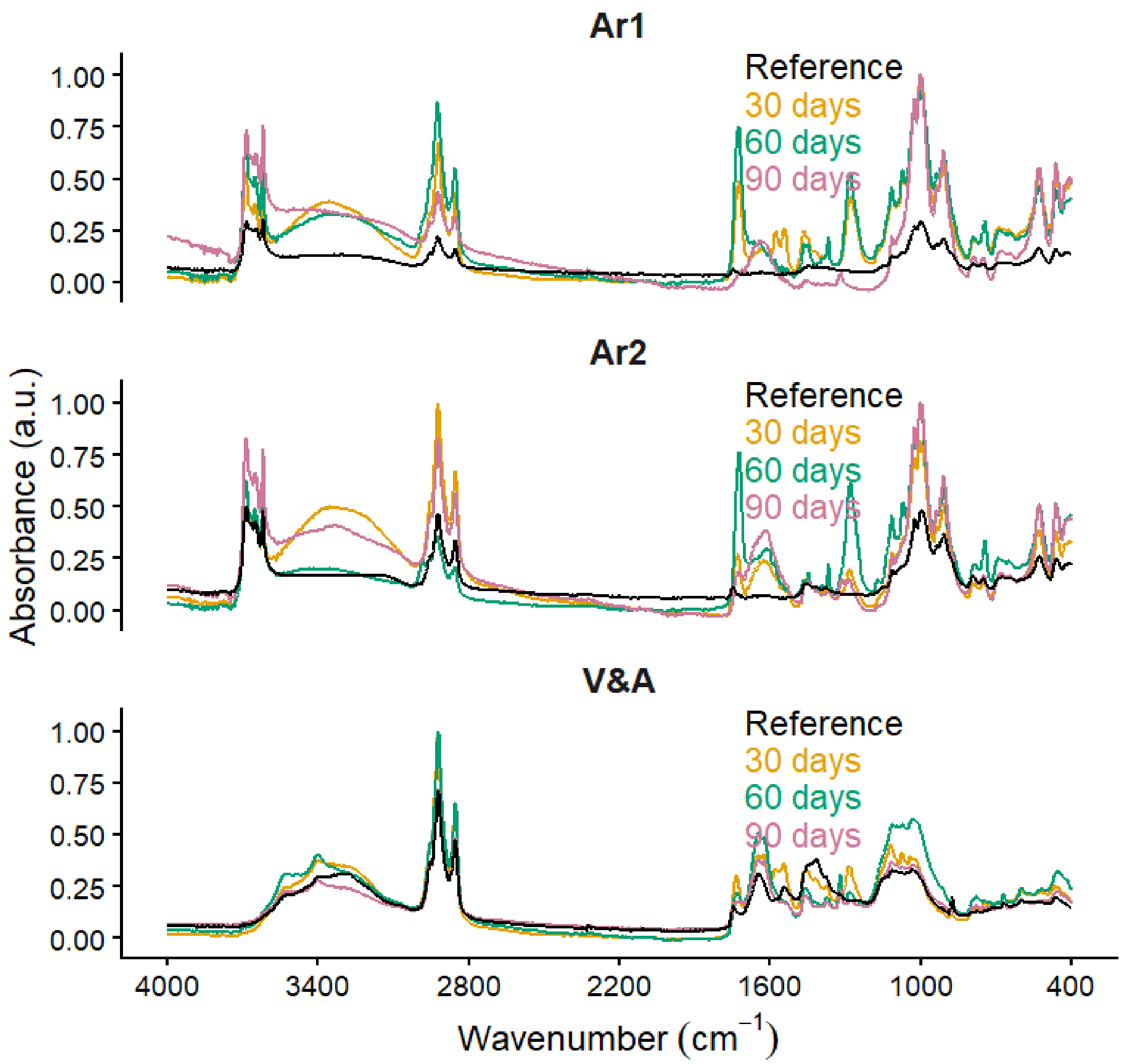

Figure S4: FTIR spectra of three PMs before (reference) and after ageing (30-60-90 days). The artificial PM formulations exhibit increased O-H stretching (3000-3500 cm^{-1}) and transient bands near 1725 and 1280 cm^{-1} under acidic conditions, indicating surface modification following exposure to acetic acid, whereas the V&A PM shows comparatively limited changes. The normalised data is presented as an average of three samples repeats with each sample unit measured three times (N=9).

3 SI.3: Mass change data

Table S1: Table of mass change (%) for Clear, V&A, Ar1 and Ar2 samples over 90 days of artificial ageing, under acidic and non-acidic conditions. The data is presented as the average of three measurements performed on three sample repeats with their standard deviation (N=9).

Sample	Time (days)	Mass change (%)	STD.dev
Clear NoAcOH	30	-9.8	0.3
Clear NoAcOH	60	-10.6	0.2
Clear NoAcOH	90	-11.7	0.0
Clear AcOH	30	-27.5	1.5
Clear AcOH	60	-39.5	0.3
Clear AcOH	90	-45.8	14.1
V&A NoAcOH	30	-9.0	0.7
V&A NoAcOH	60	-11.8	0.5
V&A NoAcOH	90	-10.4	1.1
V&A AcOH	30	-12.9	0.7
V&A AcOH	60	-24.5	0.4
V&A AcOH	90	-27.8	0.7
Ar1 NoAcOH	30	-4.8	0.4
Ar1 NoAcOH	60	-11.5	0.1
Ar1 NoAcOH	90	-10.6	0.6
Ar1 AcOH	30	-9.6	0.7
Ar1 AcOH	60	-26.9	1.0
Ar1 AcOH	90	-37.1	1.6
Ar2 NoAcOH	30	-8.8	0.6
Ar2 NoAcOH	60	-13.0	2.6
Ar2 NoAcOH	90	-11.4	0.8
Ar2 AcOH	30	-9.8	0.6
Ar2 AcOH	60	-32.8	3.0
Ar2 AcOH	90	-40.4	0.5

4 SI.4: Colour change data

Table S2: Table of colour change (a.u.) for Clear, V&A, Ar1 and Ar2 samples over 90 days of artificial ageing, under acidic and non-acidic conditions. The data is presented as the average of three measurement performed on three sample repeats with their standard deviation (N=9).

Sample	Time (days)	ΔE_{2000} (a.u.)	STD.dev
Clear NoAcOH	30	1.7	0.2
Clear NoAcOH	60	3.0	0.4
Clear NoAcOH	90	4.1	0.1
Clear AcOH	30	5.8	0.5
Clear AcOH	60	20.0	0.7
Clear AcOH	90	21.0	0.7
V&A NoAcOH	30	5.4	1.0
V&A NoAcOH	60	5.7	0.6
V&A NoAcOH	90	7.4	0.7
V&A AcOH	30	13.8	1.8
V&A AcOH	60	22.3	2.1
V&A AcOH	90	26.5	2.6
Ar1 NoAcOH	30	1.5	0.3
Ar1 NoAcOH	60	4.8	0.1
Ar1 NoAcOH	90	7.7	3.0
Ar1 AcOH	30	7.5	1.8
Ar1 AcOH	60	25.2	3.0
Ar1 AcOH	90	31.2	7.4
Ar2 NoAcOH	30	5.3	1.3
Ar2 NoAcOH	60	5.4	0.5
Ar2 NoAcOH	90	10.0	1.4
Ar2 AcOH	30	10.3	4.0
Ar2 AcOH	60	39.2	13.0
Ar2 AcOH	90	50.9	4.4

5 SI.5: IR absorption bands assignment

Table S3: Absorption band assignment of CA, DEP and AcOH, with potential overlapping spectral bands during degradation (α-β-γ-δ-ε for each overlapped group). Sources: Pretsch et al. 2010 and Wypych 2023.

Wavenumber cm^{-1}	Assignment	Compound
$^{\alpha}$3500-3400(broad)	ν-OH	AcOH
$^{\alpha}$3480-3300(broad)	ν-OH	CA
$^{\beta}$3000-2900	νC-H (methyl)	AcOH
$^{\beta}$3000-2800	νC-H	CA
$^{\beta}$2980	νC-H (aliphatic)	DEP
$^{\gamma}$1760	νC=O (carbonyl)	AcOH
$^{\gamma}$1730-1740	νC=O (acetyl)	CA
$^{\gamma}$1718-1725	νC=O (ester carbonyl)	DEP
1601-1580	νC=C (aromatic)	DEP
$^{\delta}$1366	δC-H (ethyl)	DEP
$^{\delta}$1360	δC-H (methyl)	AcOH
$^{\varepsilon}$1290	νC-O	AcOH
$^{\varepsilon}$1272	νC-O (ester)	DEP
1216-1213	νC-O-C	CA
1121, 1070, 1039	νC-O-C, C-C	DEP
1030	νC-O-C (pyranose ring)	CA
745-750	δC-H (aromatic)	DEP
601	δC-C-C	CA

6 SI.6: IR Spectral information

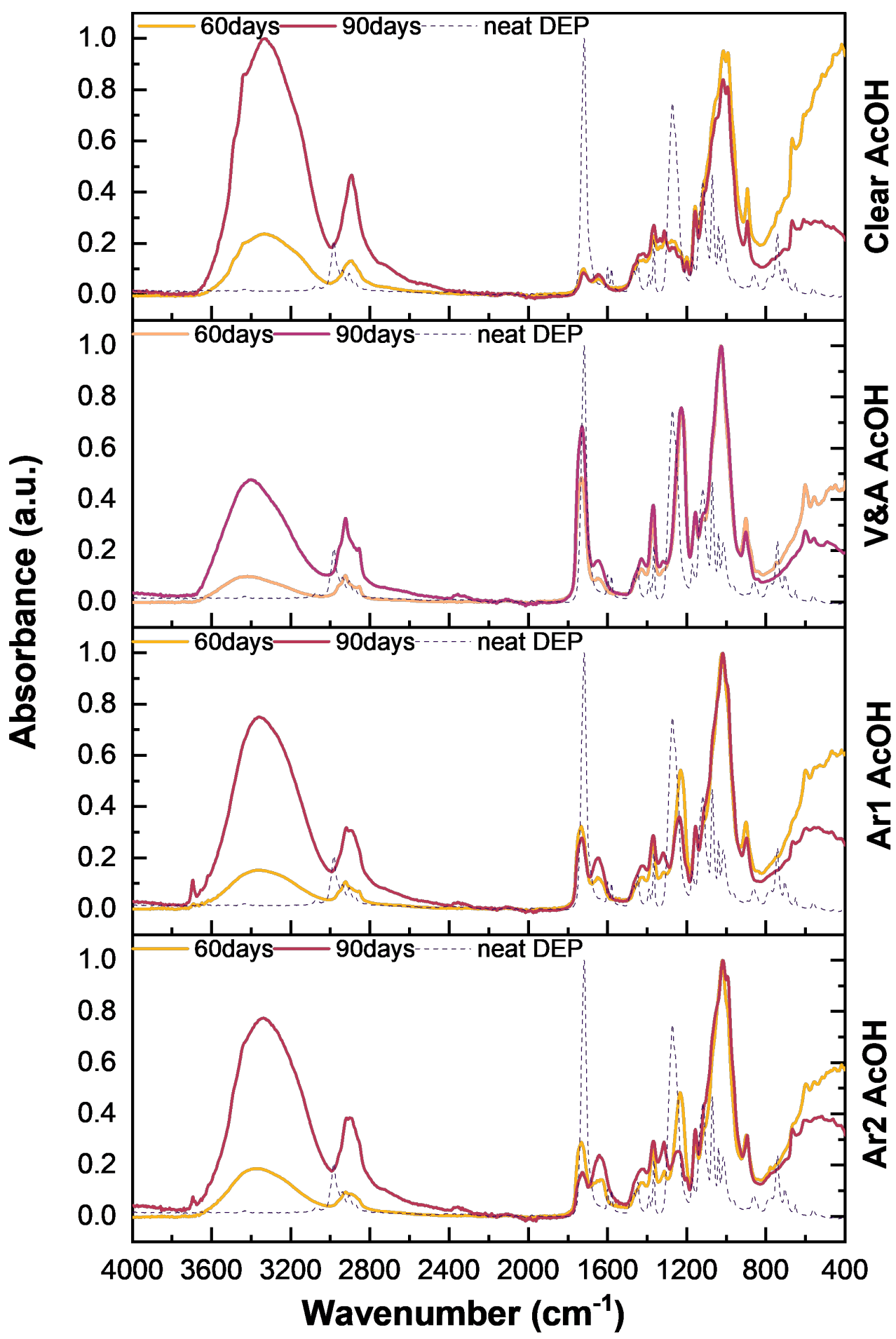

Figure S5: Infrared spectra of wiped CA samples exposed to acetic acid vapour for 60 and 90 days, alongside crude DEP. The spectra reveal overlapping absorption bands (C=O) between DEP and degrading CA, and reduced or lost spectral definition at 1030 and 601 cm^{-1}. The data is presented as the average of three measurements performed on three sample repeats, with error bars indicating the standard deviation (N=9).

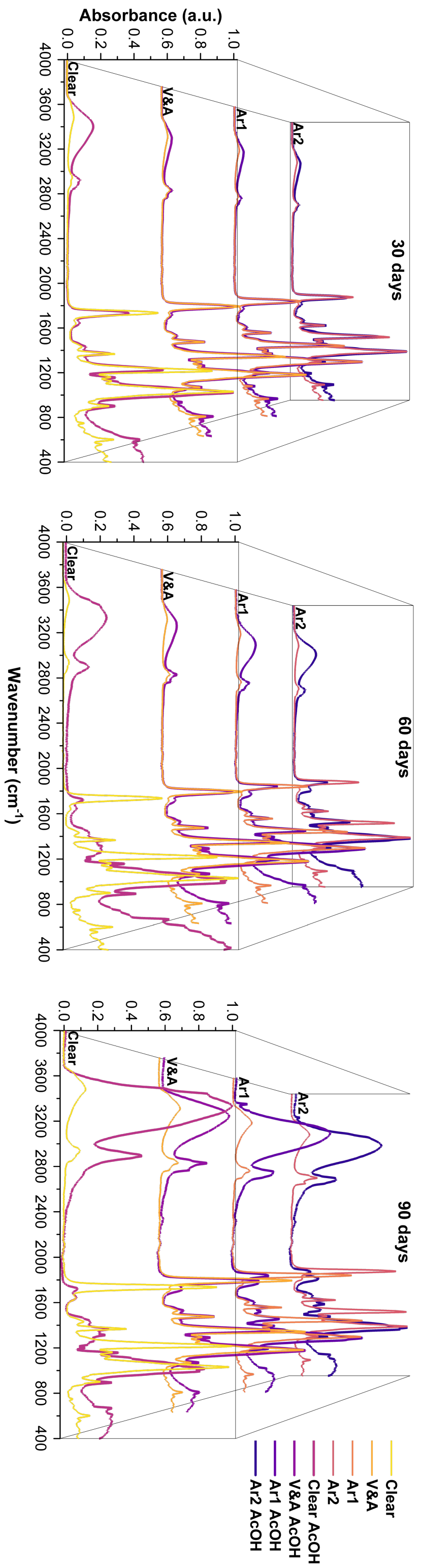

Figure S6: Infrared spectra of wiped CA samples aged for 30, 60, and 90 days under artificial ageing conditions. Spectra are organised by PM-type and grouped by treatment: non-acidic (foreground) and acidic (background) conditions for each stacked set. This arrangement highlights the influence of PM composition and acetic acid conditions on chemical changes over time. The data is presented as an average of three samples repeats with each sample unit measured three times (N=9).

7 SI.7: RSD of peak area and retention times for off-gassing acetic acid

Table S4: Relative standard deviation (RSD) of acetic acid peak area (PA) and retention times (RT) from whole CA samples [N=3] over artificial ageing (68 °C, 81% RH, acidic and not acidic conditions).

Sample	RSD[%] of PA[a]	RSD[%] of RT [a]	RSD[%] of PA[b]	RSD[%] of RT [b]	RSD[%] of PA[c]	RSD[%] of RT [c]
Clear	49.9	1.0	26.8	0.2	84.5	0.3
V&A	19	0.6	3.7	0.4	24.9	0.8
Ar1	12.4	0.6	43.6	0.8	11.4	0.3
Ar2	4.0	0.5	21.5	0.2	132.0	0.8
Clear AcOH	15.7	1.1	37	1.8	32.4	1.4
V&A AcOH	7.9	0.4	19.3	0.8	14.8	0.6
Ar1 AcOH	3.4	0.3	29.6	1.5	37.9	1.6
Ar2 AcOH	22.7	1.5	23.4	1.1	59.8	2.1

[a] 30-day, [b] 60-day, [c] 90-day of artificial ageing. Three sample repeats (same type of PM, ageing time, acidic/non-acidic condition) were run in a sequence on the same day.

Table S5: Comparison of the average relative standard deviation (RSD) for the acetic acid peak area [N=3] and the inter-day MISA peak area [N=2], with an average acquisition of three days per sample category (30/60/90-day), where each replicate was completed within the day.

Date of acquisition	RSD[%] MISA of toluene and benzene	Sample ID	RSD[%] sample
17-19 March 2025	9.6 - 17.9	**90-day** Clear	84.5
		V&A	24.9
		Ar1	11.4
		Ar2	132.0
22-25 March 2025	8.7 - 22.4	**90-day AcOH** Clear	32.4
		V&A	14.8
		Ar1	37.9
		Ar2	59.8
25-28 March 2025	21.9 - 29.4	**30-day** Clear	49.9
		V&A	19.0
		Ar1	12.4
		Ar2	4.0
30 March-2 April 2025	8.7 - 12.6	**30-day AcOH** Clear	15.7
		V&A	7.9
		Ar1	3.4
		Ar2	22.7
2-7 April 2025	22.8 - 40.1	**60-day** Clear	26.8
		V&A	3.7
		Ar1	43.6
		Ar2	21.5
7-9 April 2025	22.9 - 39.3	**60-day AcOH** Clear	37.0
		V&A	19.3
		Ar1	29.6
		Ar2	23.4

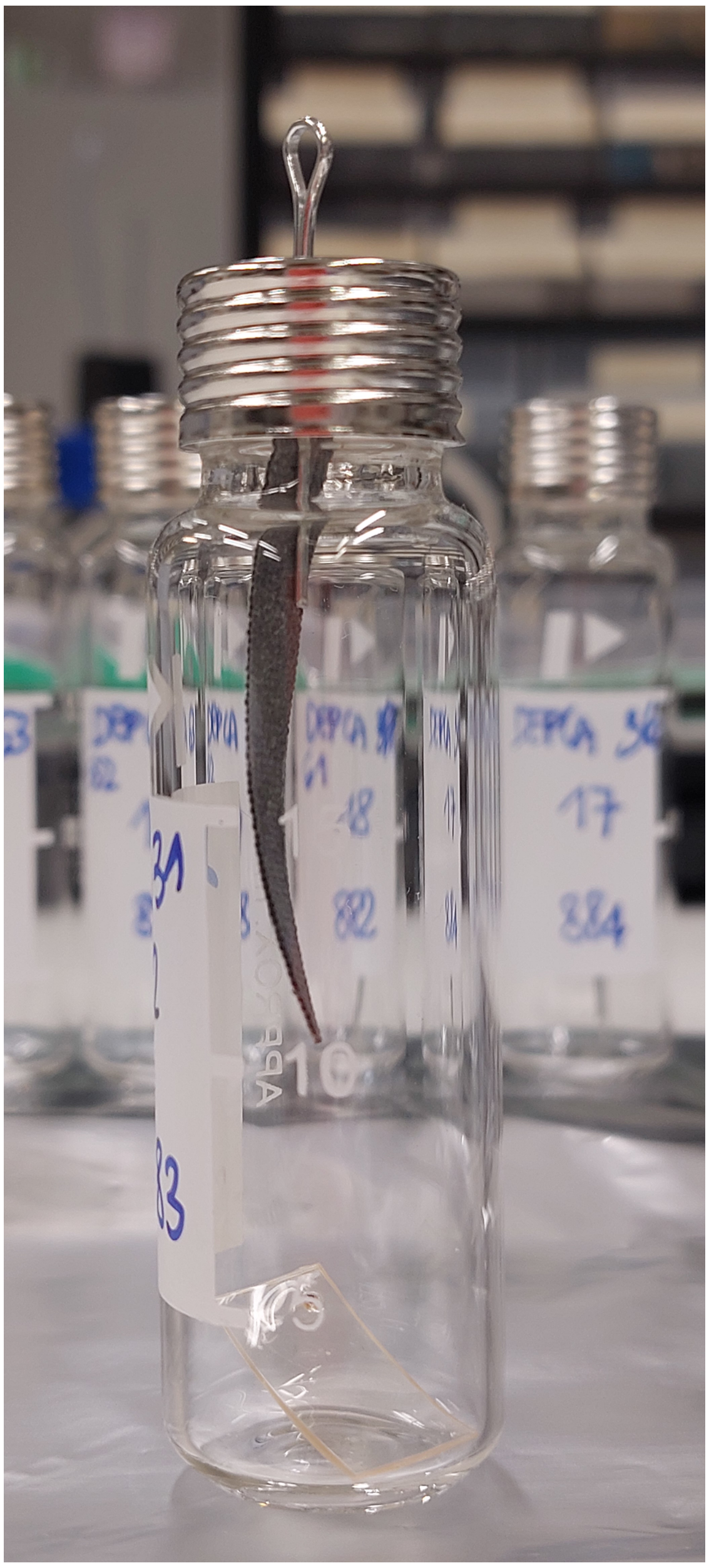

Figure S7: 20 mL vial containing CA sample and PDMS-HLB membrane ready for headspace sampling.

8 SI.8: MISA standard

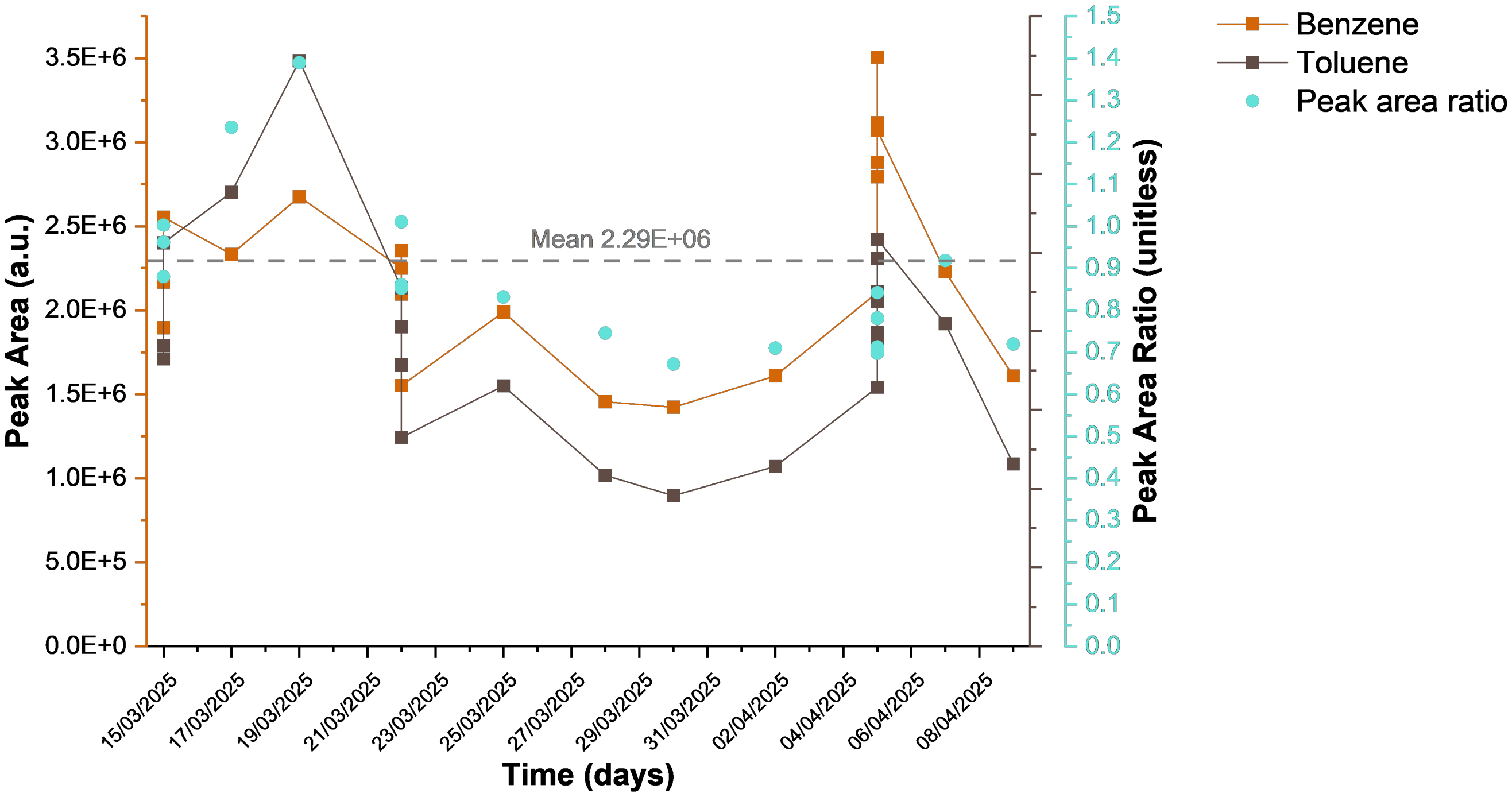

Figure S8: Variation in peak areas for the two selected external standard compounds over 25 days of acquisitions. The peak area ratio was calculated dividing the area of the benzene (retention time 16.1 min) by the area of the toluene (20.4 min), tracking intra- and inter-day measurements to account for variability and repeatability.

Table S6: Variation in the peak area and retention times (RSD) from repeated analyses (inter- and intra-day) of MISA external standard. Standard measurements were conducted between 15 March and 9 April 2025.

Analyte	Date	Inter/intra-day	RSD[%] of peak area	RSD[%] of retention time
Benzene	17-19 March 2025	Inter-day	9.6	0.3
Toluene	17-19 March 2025	Inter-day	17.9	0.3
Benzene	22-25 March 2025	Inter-day	8.7	0.1
Toluene	22-25 March 2025	Inter-day	22.4	0.1
Benzene	25-28 March 2025	Inter-day	21.9	0.1
Toluene	25-28 March 2025	Inter-day	29.4	0.1
Benzene	30 March-2 April 2025	Inter-day	8.7	0.0
Toluene	30 March-2 April 2025	Inter-day	12.6	0.0
Benzene	2-7 April 2025	Inter-day	22.8	0.1
Toluene	2-7 April 2025	Inter-day	40.1	0.1
Benzene	7-9 April 2025	Inter-day	22.9	0.1
Toluene	7-9 April 2025	Inter-day	39.3	0.1
Benzene	15 March 2025	Intra-day (3 repeats)	15.0	0.0
Toluene	15 March 2025	Intra-day (3 repeats)	19.3	0.0
Benzene	22 March 2025	Intra-day (4 repeats)	17.3	0.1
Toluene	22 March 2025	Intra-day (4 repeats)	21.8	0.1
Benzene	5 April 2025	Intra-day (5 repeats)	9.6	0.0
Toluene	5 April 2025	Intra-day (5 repeats)	12.6	0.0
